# Enhancement of Activity and Thermostability of Keratinase From *Pseudomonas aeruginosa* CCTCC AB2013184 by Directed Evolution With Noncanonical Amino Acids

**DOI:** 10.3389/fbioe.2021.770907

**Published:** 2021-10-18

**Authors:** Xianchao Pan, Jian Yang, Peijuan Xie, Jing Zhang, Famin Ke, Xiurong Guo, Manyu Liang, Li Liu, Qin Wang, Xiaowei Gao

**Affiliations:** ^1^ School of Pharmacy, Southwest Medical University, Luzhou, China; ^2^ Department of Pharmacy, The Affiliated Hospital of Southwest Medical University, Luzhou, China; ^3^ Department of Chemistry, Zhejiang University, Hangzhou, China

**Keywords:** metallopeptidase, keratinase, genetic code expansion, noncanonical amino acid, protein engineering, directed evolution, *Pseudomonas aeruginosa*, thermostability

## Abstract

A keratinase from *Pseudomonas aeruginosa* (KerPA), which belongs to the M4 family of metallopeptidases, was characterised in this study*.* This enzyme was engineered with non-canonical amino acids (ncAAs) using genetic code expansion. Several variants with enhanced activity and thermostability were identified and the most prominent, Y21pBpF/Y70pBpF/Y114pBpF, showed an increase in enzyme activity and half-life of approximately 1.3-fold and 8.2-fold, respectively. Considering that keratinases usually require reducing agents to efficiently degrade keratin, the Y21pBpF/Y70pBpF/Y114pBpF variant with enhanced activity and stability under reducing conditions may have great significance for practical applications. Molecular Dynamics (MD) was performed to identify the potential mechanisms underlying these improvements. The results showed that mutation with pBpF at specific sites of the enzyme could fill voids, form new interactions, and reshape the local structure of the active site of the enzyme.

## Introduction

The increasing consumption of chicken has led to the yearly production of millions of tons of feathers, which are the main by-products of poultry production (Li, 2019). The disposal of the vast amounts of feathers produced directly to the environment would be far beyond the capacity of nature to decompose them in a timely manner and may facilitate the growth and outbreaks of various pathogenic microorganisms. Furthermore, this manner of feather disposal could seriously threaten both economic animal and human health (Cavello et al., 2012), whereas burning feather waste is not encouraged because it causes air pollution. Considering that feathers are composed of > 90% keratin protein, they could be a cheap and sustainable protein source if an appropriate approach to their digestion could be developed.

However, keratin is one of the “hard-to-degrade” proteins, because of the high degree of disulphide bonds cross-linking, hydrophobic interactions, and hydrogen bonds in its structure (Ghaffar et al., 2018). Traditionally, feathers are processed into feather meal using chemical methods characterised by the use of high pressure and temperature. Chemical treatment is expensive and can reduce the value of feather hydrolysates by destroying some critical Amino Acids (Hassan et al., 2020). Keratin is resistant to many common proteases such as pepsin, papain, and trypsin, but can be degraded by keratinases, which are a specific class of proteolytic enzymes (Ghaffar et al., 2018). Most of the keratinases identified so far belong to the subtilisin-like serine protease subfamily (S8A: subtilases), or thermolysin-like metalloproteases family (M4). The members in S8A subfamily have a catalytic triad consisted by Asp, His and Ser residues, and most of them show nonspecific endopeptidase activity with a preferred neutral-mildly alkaline pH. The active site of the members in M4 metalloproteases family contained a single, catalytic zinc ion, and most of them are endopeptidases active at neutral pH without strictly substrate specificity (Rawlings et al., 2009).

Keratinases are widespread in many microorganisms including bacteria, actinomycetes, and fungi. Biological hydrolysis of keratin by keratinases has been considered an environmentally friendly technology, and the resulting hydrolysates can be used in animal feed supplements, edible films, and organic fertilisers (Hassan et al., 2020). The degradation of keratin in an enzymatic hydrolysis process involves two steps, namely sulfitolysis and proteolysis, which are achieved by reducing agents and keratinases, respectively (Yi et al., 2020). In addition to the treatment of keratin-rich waste, keratinases have been successfully used in many other industries including detergent production, fuel, fertiliser, cosmetics, leather, paper, food, feed, and pharmaceutical industries (Hassan et al., 2020). New applications of keratinases have also been discovered such as in the treatment of calluses, acne, and psoriasis, and the hydrolysis of amyloid fibrils related to Alzheimer’s disease and prion proteins (Johnson, 2017; Hassan et al., 2020). Thus, keratinases are versatile proteolytic enzymes and their investigation has both theoretical and economic significance.

Keratinases with high activity and thermostability are highly desirable for industrial applications and those isolated from nature usually cannot fully meet the industrial requirements (Hassan et al., 2020). Consequently, protein engineering is needed to improve the enzymatic properties of specific enzymes. Notably, protein engineering techniques, including rational design and directed evolution, mainly rely on mutual mutagenesis of the 20 canonical amino acids (cAAs) (Pagar et al., 2021). The limited building blocks of only 20 cAAs inevitably restrict the improvement of protein properties during protein engineering (Pagar et al., 2021). The function and properties of enzymes are determined by the composition and side chain interactions of the AAs, and the introduction of various noncanonical amino acids (ncAAs) into the field of protein engineering could provide more building blocks for the enzyme molecules (Pagar et al., 2021). Furthermore, this would create new interactions between the side chains of the residues and form new local molecular structures of enzymes. Consequently, enhanced activity and stability and even new functions could be generated by ncAA-based protein engineering (Pagar et al., 2021).

Genetic code expansion is a technique that can be used to site-specifically incorporate ncAAs into target proteins *in vivo* with high fidelity and efficiency (Xiao and Schultz, 2016). In genetic code expansion, a heterogeneous and orthogonal tRNA/synthetase pair is needed to insert ncAAs of interest in response to a nonsense or frameshift codon in the target protein gene. More than 200 ncAAs with different functional groups have been genetically encoded in prokaryotic, eukaryotic, and even animal cells (Chin, 2014). To date, the incorporation of ncAAs into proteins has been used in many biological fields, including to explore biological processes, probe protein structures and functions, design fluorescent protein probes, construct genetically modified organisms, capture transient protein-protein interactions, and enhance protein drugs. Recently, genetic code expansion has been used in enzyme engineering and evolution, and ncAAs have been shown to improve enzyme properties in some cases (Pagar et al., 2021).

*Pseudomonas aeruginosa* has been reported to be a keratinase producing bacterium, with high keratin hydrolysing ability (Lin et al., 2009a). *P. aeruginosa* is a Gram-negative bacterium that easily causes pneumonia in vulnerable hosts such as postoperative patients and those with cancer, burns, and cystic fibrosis (Lin et al., 2009c). Because *P. aeruginosa* is an opportunistic pathogen, the utilisation of this strain in the feather processing industry has a high risk and its keratinases need to be expressed in heterologous hosts. The keratinase gene, *KerPA*, isolated from *P. aeruginosa,* has been successfully expressed in *Escherichia coli*, *Bacillus subtilis*, and *Pichia pastoris* intracellularly or extracellularly (Lin et al., 2009a; c; b). The determination of the properties of the enzyme showed that it hydrolyses a broad range of protein substrates including insoluble proteins such as feather collagen and elastin. In this study, the stabilising factors in KerPA were investigated, and the enzyme was evolved with ncAAs using genetic code expansion.

## Material and Methods

### Material

The 2 × Taq PCR MasterMix and KOD-Plus-Neo DNA polymerase for the Polymerase Chain Reaction (PCR) were purchased from Beijing Solarbio Science and Technology Co., Ltd (Beijing, China) and Toyobo Co., Ltd (Osaka, Japan), respectively. The Fast-Digest restriction enzymes and EasyGeno Assembly cloning kit used for molecular cloning were obtained from Thermo Fisher Scientific (Rockford, IL, United States) and Tiangen Biotech (Beijing, China), respectively. The *P. aeruginosa* strain was obtained from the China Centre for Type Culture Collection (CCTCC, ID: AB2013184). The plasmids pULTRA-CNF (ID: 48215) and pEVOL pBpF (ID:31190) were purchased from Addgene (Watertown, MA, United States). The primers were designed in our laboratory and synthesised by Sangon Biotech Co., Ltd (Shanghai, China). The ncAAs were purchased from Amatek Scientific Co., Ltd (Suzhou, China) and azocasein was obtained from Sigma-Aldrich (St. Louis, MO, United States). All other chemicals and reagents used were of analytical grade.

### Bacterial Strains and Growth Conditions

*P. aeruginosa* was grown in Luria-Bertani (LB) medium at 30°C and used for *KerPA* gene amplification and *E. coli* DH5α was used for molecular cloning. The *E. coli* Origami™ (DE) pLysS strain was used for intracellular protein expression, whereas *E. coli* C321ΔA. Exp.T7, which was generated by the integration of T7 RNA polymerase into the genome of *E. coli* C321ΔA. Exp, was used for extracellular protein expression. Unless otherwise indicated, the *E. coli* strains were cultured at 37°C in LB medium supplemented with kanamycin (30 μg/ml), spectinomycin (50 μg/ml), chloromycetin (34 μg/ml) or ampicillin (50 g/ml) as needed.

### Efficiency of Incorporating ncAAs Into Proteins

The nucleotide sequence of KerPA has been submitted to the GenBank nucleotide sequence database under accession no. OK336486. The sequences of the primers used for amplification of the target DNA fragment and gene mutagenesis are shown in Supplementary Table S1. The *KerPA* gene was amplified from the genome of *P. aeruginosa* using colony PCR with primers P1 and P2, and cloned into *Nde*I and *Xho*I sites of the plasmid pET22b to construct the intracellular expression plasmid pET22b-*KerPA* using the EasyGeno Assembly cloning kit, according to the manufacturer’s instructions. To construct the extracellular expression plasmid, the *KerPA* gene amplified using primers P3 and P4 was cloned into the *Nco*I and *Hind*III sites of the plasmid pET26b to generate the recombinant plasmid pET26b-*kerPA*.

The point mutants were constructed using the QuikChange Site-Directed Mutagenesis (SDM) method with plasmid pET22b-*KerPA* or pET26b-*kerPA* as the template. The plasmid pET22b-*GFPY151TAG* harbouring a gene encoding Green Fluorescent Protein (GFP) with an amber mutation at the permissive site Tyr-151 was used to test the efficiency of incorporating NCAAs into proteins using plasmid pULTRA-CNF and pEVOL-pBpF as described previously [5]. Detailed information on the plasmids used in this study is presented in Supplementary Table S2.

### Determination of Incorporation Efficiency of ncAAs

The pET22b-*GFPY151TAG* and pULTRA-CNF or pEVOL-pBpF plasmids were co-transformed into *E. coli* Origami™ (DE) pLysS competent cells. The recombinant cells were grown in LB medium supplemented with kanamycin, ampicillin, and spectinomycin or chloromycetin at 37°C. After the optical density at a wavelength of 600 nm (OD_600_) value reached 0.4,1 mM ncAAs and 0.4 mM Isopropyl-β-D-1-Thiogalactopyranoside (IPTG) were added to the culture and shaken at 30°C for another 16 h. After expression, 1 ml of each sample was collected by centrifugation at 5,000×*g* for 10 min and washed three times with sterile phosphate buffer (PB, pH 7.5). Then, the cells were resuspended in PB and the in-cell fluorescence was measured using a plate reader (excitation/emission wavelength [E_x_/E_m_] = 488 nm/513 nm) in a 96-well plate.

### Protein Expression and Purification

Unless otherwise indicated, the Wild-Type (WT) and non-ncAA-containing variants were expressed intracellularly using *E. coli* Origami™ (DE) pLysS as the expression host. In contrast, the ncAA-containing variants were expressed extracellularly using *E. coli* C321ΔA. exp.T7 as the expression host. Cells harbouring the corresponding plasmids were cultured in 200 ml LB medium at 37°C until the OD_600_ value reached 0.6. Furthermore, 0.4 mM IPTG was added to the culture, and when ncAAs containing proteins were expressed, 1 mM ncAAs was simultaneously added to the culture. The cultures were then shaken at 20°C for 40 h to induce protein expression, and then the cells or culture supernatants were harvested by centrifugation at 10,000×*g* for 10 min.

To purify the intracellularly expressed proteins, the cell pellets were washed, suspended in PB, and sonicated on ice. The resulting supernatants were collected after centrifugation and incubated at 50°C for 1 h to allow the enzyme to mature and digest the host proteins. The crude enzyme samples were finally purified using Ni^2+^-charged chelating Sepharose Fast Flow columns (GE Healthcare, Uppsala, Sweden). To purify the extracellularly expressed proteins, the culture supernatants were first precipitated using ammonium sulphate (60–80%) saturation. After centrifugation at 13,000×*g* for 10 min, the resulting precipitates were dissolved in PB and dialysed to remove the residual ammonium sulphate. The samples were incubated at 50°C for 1 h to mature the enzyme and digest the host proteins. The crude enzyme samples were purified using a Diethylaminoethyl (DEAE) anion exchange column (GE Healthcare, Uppsala, Sweden) equilibrated with 50 mM glycine-sodium hydroxide (NaOH) buffer (pH 10.5). After purification, the samples were dialyzed in PB and the purified enzyme samples were concentrated using a Microcon YM-3 centrifugal filter (Millipore, Bedford, MA, United States) when needed.

### Protein Quantification and Electrophoresis

The proteins were quantified using the Bradford method with Bovine Serum Albumin (BSA) as the standard. The proteins were separated using Sodium Dodecyl Sulphate-Polyacrylamide Gel Electrophoresis (SDS-PAGE) with a Tris-glycine buffer or tris-tricine buffer (tricine–SDS-PAGE) system using a 12% polyacrylamide gel. The protein samples for electrophoresis were prepared as follows: the proteins were first precipitated with 200 g/L Trichloroacetic acid (TCA), washed with ice-cold acetone, solubilized in a loading buffer containing 8 M urea with or without 5 mM DL-dithiothreitol, and then electrophoresed using the gel followed by staining with Coomassie brilliant blue R250.

### Enzyme Activity Assay

Unless otherwise indicated, the enzyme activity was assayed using a 400 µL reaction mixture containing 2.5 g/L azocasein and 200 µL of the enzyme sample at 50°C for 10 min. After cooling on ice, the reaction was terminated by adding 400 µL TCA (w/v) and incubating at room temperature for 15 min. After centrifugation at 13,000 × *g* for 10 min, the absorbance of the supernatant was determined at 335 nm using a plate reader (Multiskan GO; Thermo Fisher Scientific, Franklin, MA, United States). One unit of activity was defined as the amount of enzyme required to increase the absorbance at a wavelength of 335 nm (A_335_) value by 0.01/min.

The activity of the enzyme on other soluble and insoluble protein substrates was determined for 1 h at 50°C in a reaction mixture (600 μL) containing 10 μg/ml enzyme and 30 mg of each substrate in PB. An equal volume of 400 g/L TCA was added to the reaction mixture to terminate the reaction. After centrifugation at 13,000×*g* for 10 min, the absorbance of the supernatant was measured using a plate reader, as described previously (Yi et al., 2020). The kinetic parameters of KerPA and its variants were assayed in PB buffer at 50°C using the synthetic substrate N-succinyl-Ala-Ala-Pro-Phe-pNA. The experiments were carried out as described previously (Yi et al., 2020), and the *K*_*m*_ and *k*_*cat*_ values were calculated using the Table Curve 2D software (Jandel Scientific, version 5.0).

### Screening of the ncAAs Variants

The pET26b-*kerPA* plasmid carrying different TAG mutagenesis was co-transformed with the orthogonal plasmids into *E. coli* C321ΔA. Exp.T7. The cells were cultured in a deep 96-well plate at 37°C until the OD600 value reached 0.6. Protein expression was induced by adding 1 mM ncAAs, 0.4 mM IPTG, and 2 g/L arabinose as needed. The plates were shaken for another 40 h at 20°C. After protein expression, the plates were centrifuged at 5,000×*g* for 10 min, and the culture supernatants were collected. To compare the activity of the crude enzyme samples, 200 µL of the resulting culture supernatant of each sample was assayed for azocaseinolytic activity as described above. The resulting culture supernatant (200 µL) of each sample was incubated at 60°C for 1 h and the residual activity was determined to compare the thermostability of the crude enzyme samples.

### Mass Spectrometry Analysis

The experiments for the Mass Spectrometry (MS) analysis were performed as previously described (Yi et al., 2020). In brief, the protein samples in PB were pre-treated using buffer exchange with the native buffer (10 mM ammonium acetate, pH 6.6). High-resolution MS was performed using a Q-Exactive HF-X mass spectrometer (Thermo, United States) coupled with a Waters ACQUITY Ultraperformance Liquid Chromatography (UPLC) system. The protein mass was deconvoluted by extraction of the total ion count across the entire area of protein elution using Thermo Xcalibur 4.0 software (Thermo, United States).

### Homology Modelling and Molecular Dynamics Analysis

The structure of the WT KerPA protein was constructed using homology modelling with the Swiss-model server (https://swissmodel.expasy.org/). The side chains of the three tyrosine residues (Y21, Y70, and Y144) were modified using the LEaP program (Salomon-Ferrer et al., 2013). The zinc ion in the catalytic site was modelled using the 12-6-4 LJ-type nonbonded extended simple point charge (SPC/E) water model (Li and Merz, 2014; Panteva et al., 2015).

The protonation states of the charged residues were assigned by p*K*a calculations at pH 7.0 using the PDB2PQR server (Dolinsky et al., 2004). All histidine residues were neutral and protonated at the *ε* nitrogen atoms and the N- and C-terminal residues of the protein were considered neutral. Each model was explicitly solvated in the rectangular periodic water box of the SPC/E model with a minimal distance of 10 Å from the protein to the box edges (Price and Brooks, 2004). The resulting system contained approximately 37,000 atoms and was used for the MD simulations.

MD simulations were performed using the Amber 18 suit of programs with the Amber ff14SB force field (Case et al., 2005; Maier et al., 2015). To remove excessive strain and bad contacts, each system was first optimised using 2500 steps of the steepest descent followed by 2500 steps of conjugate gradient energy minimisations and a harmonic force of 500 kcal mol^−1^·Å^−2^ applied to the protein. Next, 5000 steps of the steepest descent followed by 5000 steps of conjugate gradient energy minimisations were performed to relax the entire system in the absence of harmonic restraints. Then, each system was gradually heated from 0 to 333 K by 200 ps constant volume dynamics, followed by 200 ps constant temperature dynamics at 333 K with the protein constrained using a harmonic force of 50 kcal mol^−1^·Å^−2^ in the NVT ensemble. Finally, a 200 ns production simulation was performed in the NPT ensemble (*T* = 333 K, *P* = 1 bar) for each system. An integration time step of 2 fs was used for all simulations. A cut-off of 10 Å was used for van der Waals and electrostatic interactions and the long-range electrostatic interactions were estimated using the Particle Mesh Ewald (PME) method (Darden et al., 1993). Bond lengths involving hydrogen atoms were constrained using the SHAKE algorithm (Ryckaert et al., 1977). The Langevin thermostat was used to equilibrate the temperature with a collision frequency of 1.0 ps^−1^. The Isotropic Berendsen coupling barostat with compressibility of 4.5 × 10^−5^bar^−1^ was used to control the pressure. The Root Mean Square Deviation (RMSD) and Root Mean Square Fluctuation (RMSF) of the C_α_ atoms were calculated using the Cpptraj program (Roe and Cheatham, 2013).

### Statistical Analysis

Each of the experiments was carried out in triplicate. The data were subjected to analysis of variance with IBM Statistical Package for Social Science (version 23), and compared at a *p* < 0.05 significant level. The results were presented as mean values ± standard deviation.

## Results

### KerPA Sequence and Structural Information

**FIGURE 1:**
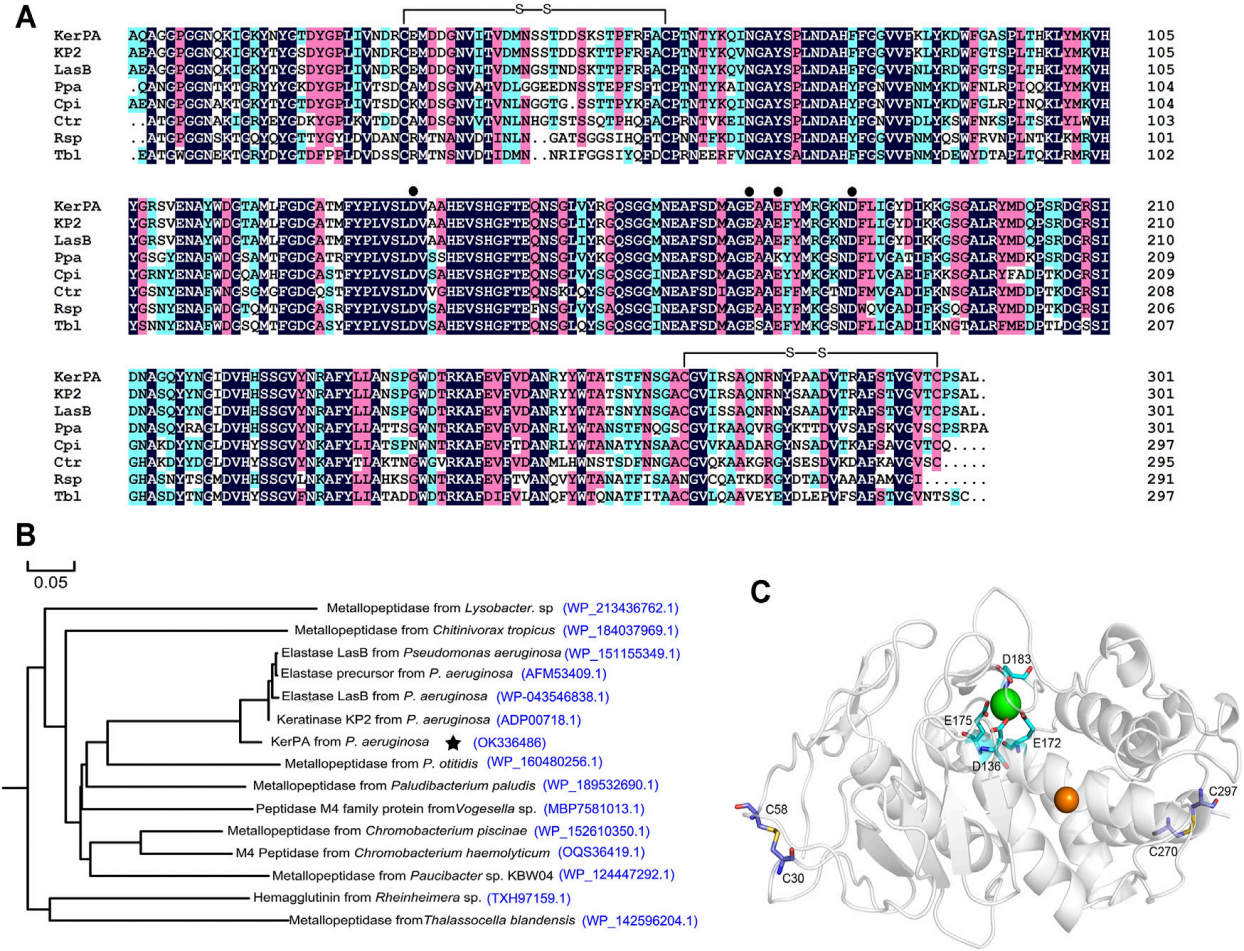
Sequence and structure analysis of keratinase from *Pseudomonas aeruginosa* (KerPA). **(A)** Multiple sequence alignment of KerPA and its homologs. Catalytic domain of KerPA from *Pseudomonas aeruginosa* isolated in this study is aligned with keratinase KP2 from *P. aeruginosa* (accession number: ADP00718.1), elastase LasB from *P. aeruginosa* (accession number: WP_043546838.1), metallopeptidase Ppa from *Paludibacterium paludism* (accession number: WP_189532690.1), metallopeptidase Cpi from *Chromobacterium piscinae* (accession number: WP_152610350.1), metallopeptidase Ctr from *Chitinivorax tropicus* (accession number: WP_184037969.1), Rsp from *Rheinheimera* sp (accession number: TXH97159.1), and metallopeptidase Tbl from *Thalassocella blandensis* (accession number: WP_142596204.1). Filled circles show calcium ion ligand residues. Two disulphide bonds are shown as black lines. Alignments were generated using clustalX2 program and coloured using DNAMAN 5.2.2 software. Conserved residues in all sequences are indicted by black background. **(B)** Phylogenomic tree of KerPA. Set of 15 keratinase proteins with high homology were selected to build the phylogenomic tree. Phylogenomic analysis was performed using MEGA 5.2 software and multiple alignment was conducted using Clustal W (default parameters). **(C)** Homology modelled structure of KerPA. Green and orange balls in protein structure represent bound calcium and zinc ions, respectively. Two disulphide bonds and ligand residues involved in calcium ion binding are shown as sticks.

The *KerPA* gene, amplified from the genome of *P. aeruginosa*, encodes a polypeptide chain with 506 AA residues, including a 23-residue signal peptide and a 174-residue N-terminal propeptide (intramolecular chaperone). The N-terminal propeptide is removed and degraded after enzyme maturation and the sequence of the mature catalytic domain of KerPA (mKerPA) was aligned with other metalloproteases from different microorganisms. As shown in Figure 1A, the sequence identities of mKerPA with KP2 and LasB from *P. aeruginosa*, Ppa from *Paludibacterium paludism*, Cpi from *Chromobacterium piscinae*, Ctr from *Chitinivorax tropicus*, Rsp from *Rheinheimera* sp., and Tbl from *Thalassocella blandensis* were 96.32, 94.98, 74.32, 74.74, 67.47, 63.16, and 61.11%, respectively.

A phylogenetic tree that revealed the evolutionary relatedness and levels of homology of mKerPA with 14 other metallopeptidases is shown in Figure 1B. The crystal structure of the solvent tolerant elastase from *P. aeruginosa* strain K was used as a template (PDB ID: 4k89.1) to generate the structural model of KerPA using the SWISS-MODEL server (Figure 1C). According to the modelled structure and the aligned sequence, two disulphide bonds and one calcium ion binding site were well conserved in the structure of KerPA (Figures 1A,C).

### Overexpression and Enzymic Properties of Recombinant KerPA

*E. coli* C321ΔA exp.T7 was generated in our laboratory by integrating T7 RNA polymerase into the genome of *E. coli* C321ΔA. Exp (Yi et al., 2021). *E. coli* C321ΔA exp was constructed by replacing all known amber UAG codons with synonymous ochre UAA codons and deleting the gene of release factor 1 (*RF1*) in the genome of *E. coli* MG1655 (Lajoie et al., 2013; Kuznetsov et al., 2017). Using *E. coli* C321.ΔA derivative strains as an expression host produced full-length proteins bearing one or multiple ncAAs with a comparable yield to those of the WT proteins.

**FIGURE 2:**
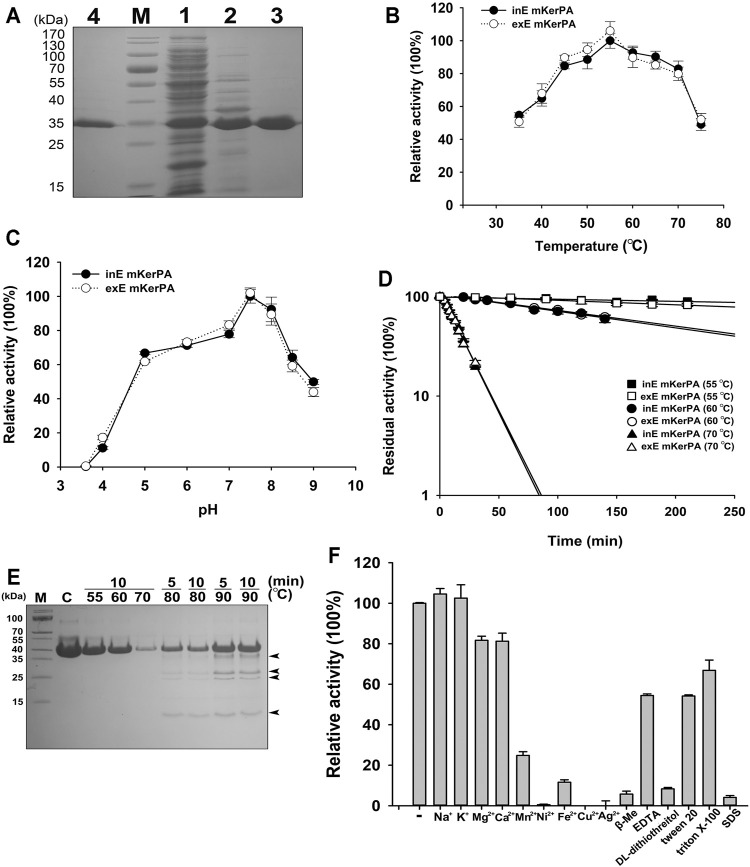
Enzymic properties of recombinant keratinase from *Pseudomonas aeruginosa* (KerPA). **(A)** SDS-PAGE analysis of purification procedures for recombinant KerBL. M, protein markers; Lane 1, supernatant of total cell extract of *Escherichia coli* Origami™ (DE)pLysS harbouring pET22b-*KerPA*; Lane 2, sample from Lane 1 after heat treatment at 50°C for 1 h; Lane 3, samples from Lane 2 purified using Ni^2+^-charged chelating Sepharose Fast Flow column; Lane 4, purified samples from culture supernatant of *E. coli* C321ΔA exp.T7 harbouring pET26b-*KerPA*. **(B)** Effect of temperature on enzymic activity of KerPA. Samples of intracellularly expressed KerPA (inE mKerPA) and extracellularly expressed KerPA (exE mKerPA) enzymes incubated with 2.5 g/L azocasein in phosphate buffer (PB, pH 7.5) at various temperatures for 10 min. Activities at an optimal temperature (55°C) were defined as 100%. **(C)** Effect of pH on enzymic activities of inE KerPA and exE mKerPA. Enzyme activity was assayed at different pH, and the highest activity was defined as 100%. **(D)** Heat inactivation profiles of inE KerPA and exE mKerPA. Enzyme samples were incubated at 55, 60, and 70°C for indicated times and assayed for residual activity using azocasein as substrate at 55°C. **(E)** SDS-PAGE analysis of samples of inE KerPA enzyme incubated at different temperatures. Autocleavage products are indicated with black arrowheads. **(F)** Effects of metal ions and chemical reagents on activity of inE KerPA. Azocaseinolytic activity of inE KerPA in the presence of different metal ions and chemical reagents was assayed after incubation at 55°C for 10 min. Activities of control group were defined as 100%. Values are means ± Standard Deviation (SD) of three independent experiments.

First, the expression of the *KerPA* gene was carried out intracellularly and extracellularly in *E. coli* C321ΔA. exp.T7. However, only the extracellularly expressed KerPA showed remarkable activity, whereas the intracellularly expressed KerPA showed no detectable activity and formed inclusion bodies in *E. coli* C321ΔA. Exp.T7 (data not shown). This may be because the disulphide bonds in KerPA could not be formed properly in the reducing cytoplasm of *E. coli* C321ΔA. Exp.T7. Thus, *E. coli* Origami™ (DE) pLysS, which is commonly used to express disulphide bond-containing proteins, was used as an intracellular expression host of KerPA. The intracellularly expressed KerPA in *E. coli* Origami™ (DE) pLysS showed remarkable activity. Both the intracellularly and extracellularly expressed KerPA could be purified to homogeneity (Figure 2A).

**FIGURE 3:**
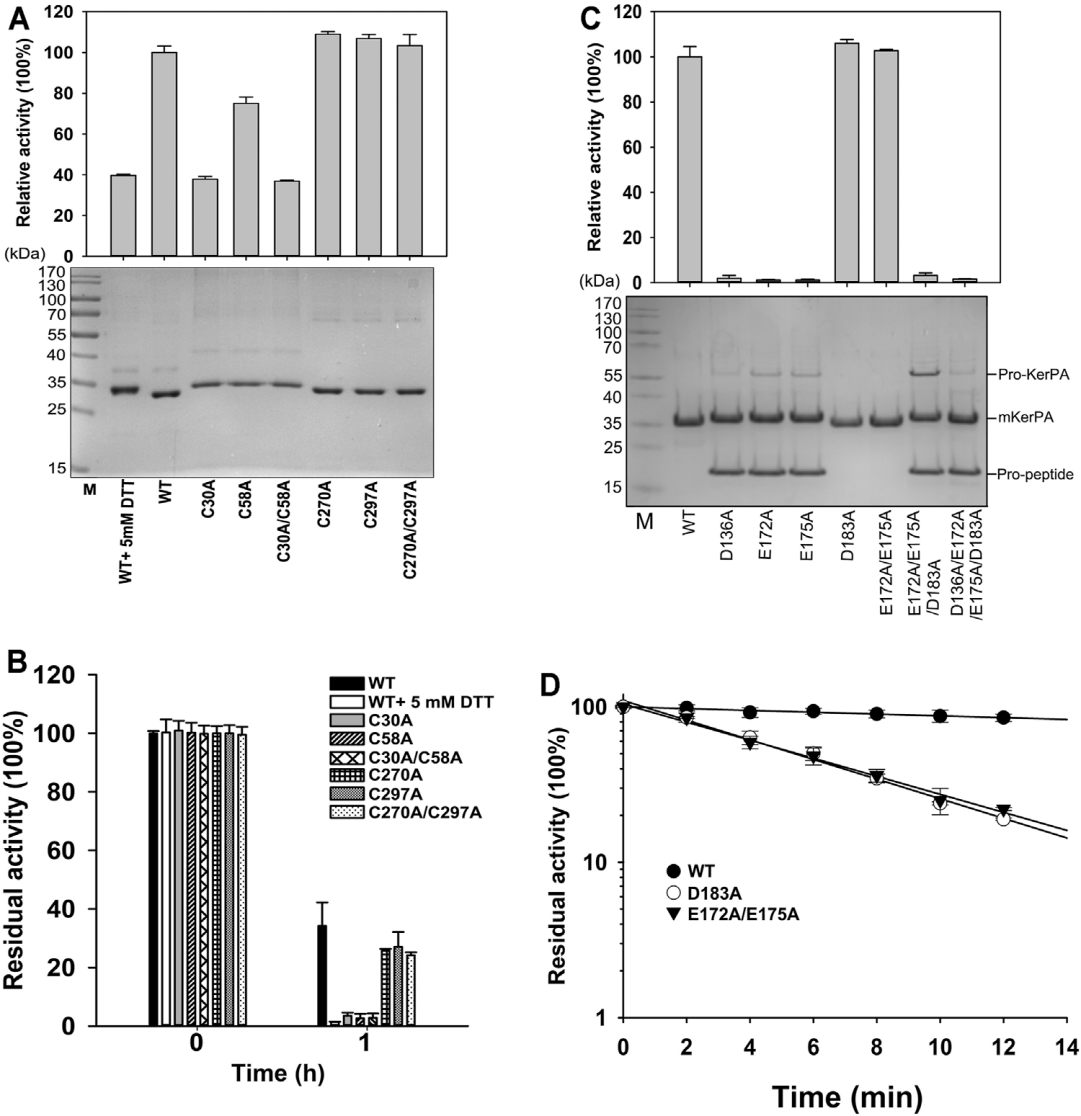
Properties of disulphide bonds and Ca^2+^ binding related variants. **(A)** Activity assays (*upper*) and SDS-PAGE analysis (*lower*) of purified samples of variant enzymes and wild-type (WT) with or without 5 mM DL-dithiothreitol. Activity was assayed at 55°C in Phosphate Buffer (PB) using azocasein as substrate, and relative activity was calculated by defining activity of WT without 5 mM DL-dithiothreitol as 100%. **(B)** Thermostabilities of variants and WT with or without 5 mM DL-dithiothreitol. Purified samples in PB were incubated at 65°C for 1 h, and residual activities were determined using azocasein as substrate. **(C)** Activity assays (*upper*) and SDS-PAGE analysis (*lower*) of purified samples of variant enzymes. Azocaseinolytic activity was assayed at 55°C in PB. Proform of keratinase from *Pseudomonas aeruginosa* (KerPA), mature enzyme, and propeptide in protein samples are shown on gel. **(D)** Heat inactivation profiles of variants. Enzyme samples were incubated at 65°C for indicated times, and residual activity was assayed using azocasein as substrate at 55°C. Values are means ± Standard Deviation (SD) of three independent experiments.

The enzymatic properties of the intracellular and extracellular mKerPA were compared, and the results suggested that the optimal temperature, optimal pH, and heat inactivation profiles were similar between the two expressed forms (Figures 2B–D). The mKerPA showed maximal activity at 55°C, and > 50% of its activity was retained over a wide range of temperatures from 30 at 70°C. The enzyme showed > 60% of its highest activity from pH 5–8.5, with an optimum pH of 7.5. According to the inactivation curves shown in Figure 3D, mKerPA was relatively stable at 55 and 60°C, but underwent denaturation, auto-degradation, or both quickly at 70°C. When incubated at 80 and 90°C, the enzyme underwent autolysis and four autocleavage products could be detected by the SDS-PAGE analysis (Figure 2E).

The effects of different metal ions and chemical reagents on the enzymatic activity of mKerPA are shown in Figure 2F. The results suggested that Na^+^ and K^+^ had little effect on enzyme activity. Mg^2+^ and Ca^2+^ slightly reduced the enzymatic activity, whereas Mn^2+^, Ni^2+^, Fe^2+^, Cu^2+^, and Ag^2+^ showed strong inhibitory effects on the enzyme activity. DL-dithiothreitol, β-mercaptoethanol (β-Me), and SDS strongly inhibited enzyme activity, whereas ethylenediaminetetraacetic acid (EDTA), Tween 20, and Triton X-100 reduced the activity of the enzyme to various extents.

### Roles of Disulphide Bonds and Ca^2+^-Binding Site in mKerPA

There are two disulphide bonds and one Ca^2+^-binding site in the KerPA structure (Figures 1A,C). In this study, the roles of these structural elements were investigated by SDM. The mKerPA migrated slightly faster under non-reducing conditions than it did under reducing conditions when subjected to SDS-PAGE, indicating that disulphide bonds were indeed present in the structure of the enzyme (Figure 3A). As shown in Figures 3A,B, both the activity and stability of the enzyme were remarkably decreased under reducing conditions, which suggested that the two disulphide bonds were critical to the function of mKerPA.

Next, the disulphide bonds were destroyed by substituting the cysteine residues with alanine, and the functions of the two disulphide bonds were investigated individually. The three C30–C58 disulphide bond-related variants C30A, C58A, and C30A/C58A showed lower activity and significantly lower stability than that of the WT. The three C270–C297 disulphide bond-related variants C270A, C297A, and C270A/C297A showed activity that was comparable to that of the WT, and their stability was only slightly lower. These results suggest that the C30–C58 disulphide bond played an important role in both the activity and stability of the enzyme, whereas the C270–C297 disulphide bond only marginally improved the stability of the enzyme.

The modelled structure of mKerPA illustrated in Figure 1C shows that four residues, D136, E172, E175, and D183, are involved in calcium ion binding. To determine the role of the Ca^2+^-binding site, a series of variants with single and multiple mutation to replace the ligand residues with alanine were constructed. Only the D183A, E172A/E173A variant showed activity that was comparable to that of the WT, and the other five variant samples showed no activity. The results of the SDS-PAGE analysis illustrated in Figure 3C shows that the purified D136A, E172A, E175A, E172A/E173A/D183A, and D136A/E172A/E173A/D183A variant samples still contained the N-terminal propeptide of KerPA. The propeptide is an inhibitor of the catalytic domain of KerPA, which might explain why the five variants did not show any activity. The incubation time was prolonged in an attempt to activate the variants, but no activity was detected, which suggests that the Ca^2+^-binding site had no effect on the enzyme folding, but affected the maturation process by hindering the degradation of the propeptide. Next, the stability of the two variants, D183A and E172A/E173A, was comparatively analysed, and their half-lives were significantly shorter than those of the WT (Figure 3D). Taken together, these results suggest that the Ca^2+^-binding site played an important role in the maturation and thermostability of the enzyme.

### Directed Evolution of KerPA With ncAAs

**FIGURE 4:**
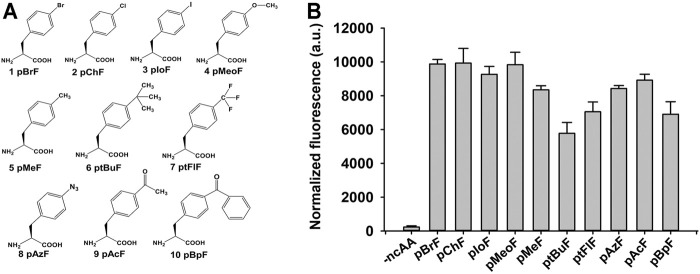
Structures and suppression efficiencies of non-canonical amino acids (ncAAs). **(A)** Structures of 10 ncAAs used in this study. **(B)** Incorporation efficiencies of different ncAAs were evaluated using Green Fluorescence Protein (GFP)-based assay. In-cell fluorescence of GFP variant Tyr151TAG was recorded using plate reader. Values are means ± Standard Deviation (SD) of three independent experiments.

The ncAAs have shown considerable versatility in the enzyme-directed evolution field [6]. There were 21 tyrosine residues in the mature form of KerPA (Supplementary Table S3). Each residue was replaced with 10 benzenoid ncAAs to construct 210 ncAAs containing variants in this study (Figure 4A). The suppression efficiency and polyspecificity of orthogonal plasmids for the 10 ncAAs in *E. coli* C321ΔA. Exp.T7 were first determined using a fluorescence-based screening method coupled with GFP expression, as described previously (Yi et al., 2020). As shown in Figure 4B, compared with the control (without ncAAs), significant in-cell fluorescence was detected in the presence of ncAAs, which indicated that the orthogonal plasmids used in this study efficiently incorporated the ncAAs into the proteins by suppressing the amber stop codon in the target gene.

**FIGURE 5:**
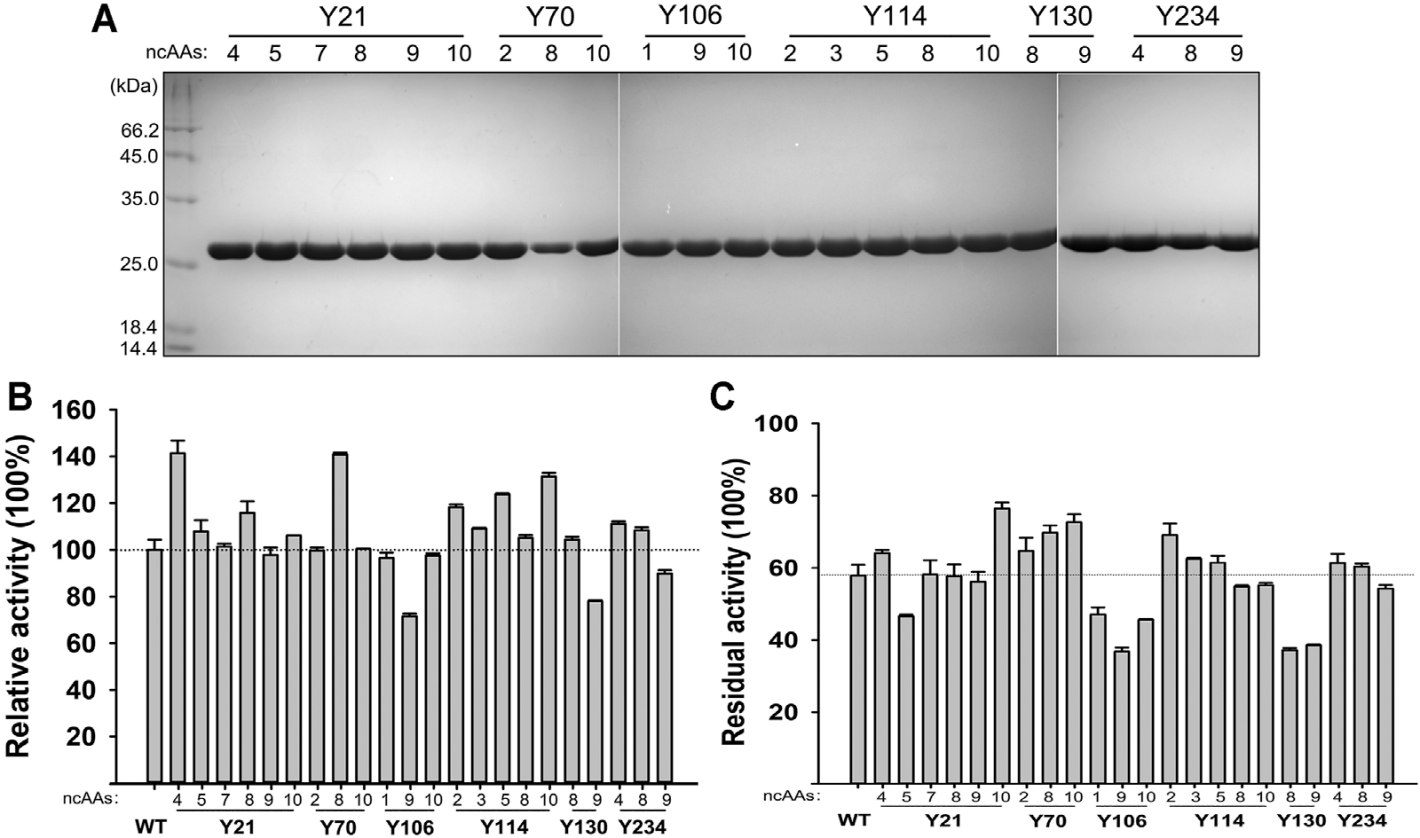
Properties of selected non-canonical amino acids (ncAAs) containing variants. **(A)** SDS-PAGE analysis of purified samples of variants. **(B)** Activities of variants. Azocaseinolytic activities of purified enzyme samples were determined at 55°C in Phosphate Buffer (PB), and relative activity was calculated by defining activity of Wild-Type (WT) as 100%. **(C)** Thermostabilities of variants. Purified enzyme samples were incubated at 65°C for 30 min, and then standard activity assay was performed using azocasein as substrate. Residual activity is expressed as percentage of original activity of each enzyme sample. Values are means ± Standard Deviations (SD) of three independent experiments.

The 210 ncAAs containing KerPA variants were expressed extracellularly in deep 96-well plates. The crude enzyme samples in the culture supernatants were assayed for activity and stability three times (Supplementary Figures S1,S2). Following a comparison of the variants with the WT, 22 variants that showed enhanced original activity or residual activity were selected, purified to homogeneity (Figure 5A), and then further analysed. Several variants showed enhanced activity and the best three, Y21pMeoF, Y70pAzF and Y114pBpF, showed a 41.3, 40.8 and 31.3% higher activity, respectively than the original enzyme (Figure 5B). In terms of thermostability, several improved variants were obtained, and the four best were Y21pBpF, Y70pAzF, Y70pBpF and Y114pChF (Figure 5C). Interestingly, two variants, Y21pMeoF and Y70pAzF, showed both enhanced activity and stability. Three pBpF-containing variants Y21, Y70 and Y114 showed increased enzyme stability and activity. To determine whether these variants have accumulative effects on the properties of the enzyme, a series of variants with double and triple mutations were constructed, and their properties were investigated in detail in subsequent study.

### Properties of Y21pBpF/Y70pBpF/Y114pBpF

**FIGURE 6:**
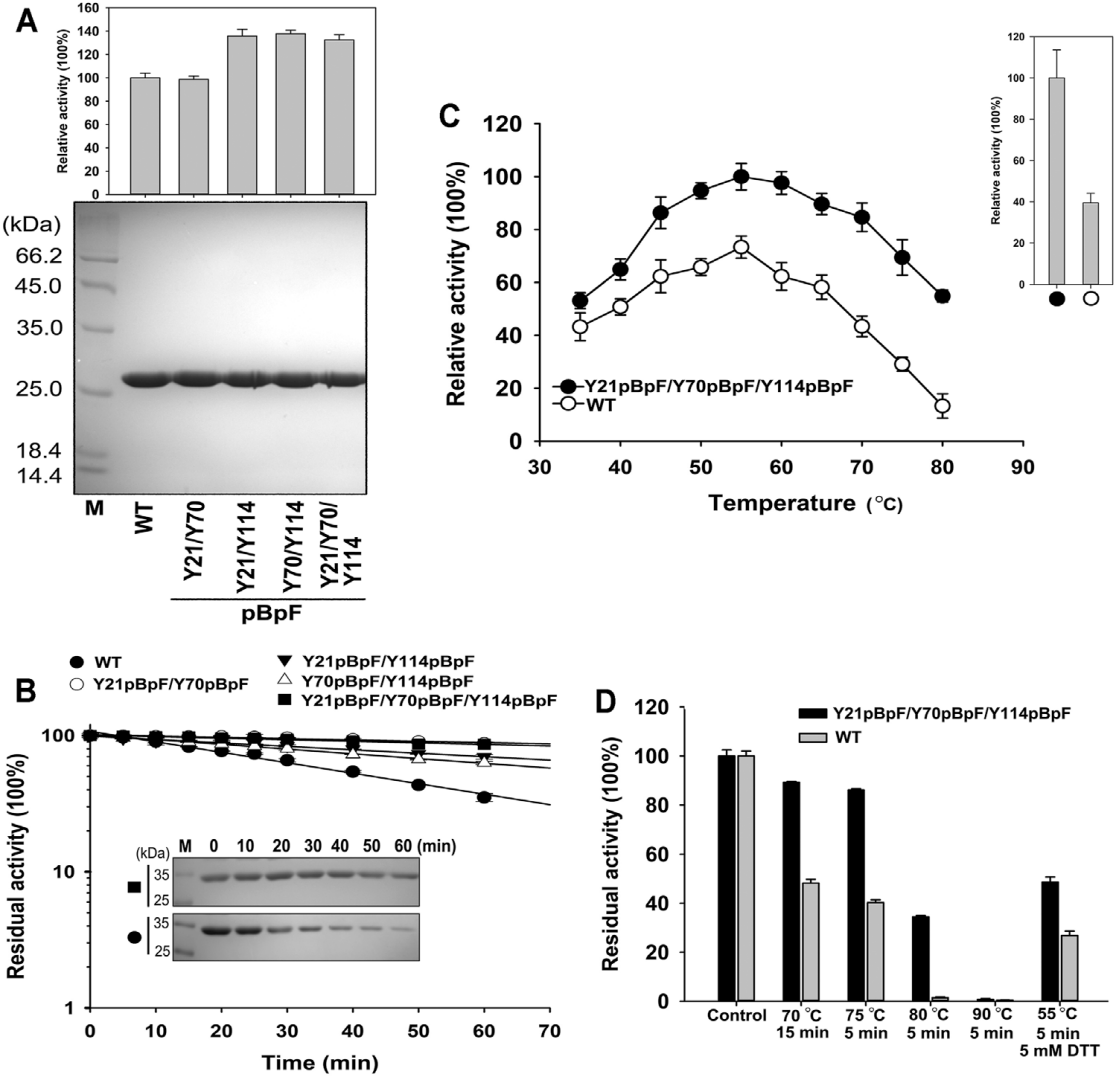
Properties of Y21pBpF/Y70pBpF/Y114pBpF variant. **(A)** Activity assays (*upper*) and SDS-PAGE analysis (*lower*) of purified samples of wild-type (WT) and its variants. Azocaseinolytic activity was assayed at 55°C in Phosphate Buffer (PB). **(B)** Heat inactivation profiles of variants. Enzyme samples were incubated at 65°C for indicated times, and assayed for residual activity using azocasein as substrate at 55°C and SDS-PAGE analysis (*inset panel*). **(C)** Activity analysis of WT and its variant at different temperatures. Enzyme samples were incubated with 2.5 g/L azocasein in PB at various temperatures for 10 min. Activity at optimal temperature of variant (55°C) was defined as 100%. Upper right panel depicts relative activity of WT and its variant in presence of 5 mM DL-dithiothreitol. **(D)** Thermostabilities of WT and its variant. Enzyme samples were incubated at different temperatures for indicated times, followed by azocaseinolytic activity assay. Values are means ± Standard Deviation (SD) of three independent experiments.

Four double or triple variants containing pBpF were expressed and purified to homogeneity (Figure 6A). The Y21pBpF/Y70pBpF variant showed an activity that was similar to that of the WT, and the other three that contained the Y114pBpF mutation showed an enhanced activity that was similar to that of the single mutation variant Y114pBpF (Figures 5B, 6A). As shown in Figure 6B, all four pBpF-containing variants had longer half-lives than that of the WT, and the double variant Y21pBpF/Y70pBpF and triple variant Y21pBpF/Y70pBpF/Y114pBpF showed the highest thermostability. Taken together, these results demonstrate that replacing Y21, Y70 and Y114 with pBpF simultaneously in mKerPA had accumulative beneficial effects on the activity and thermostability of the enzyme. The Y21pBpF/Y70pBpF/Y114pBpF variant showed improved activity and the highest thermostability among the variants and, therefore, the properties of this variant were further investigated in detail.

The azocaseinolytic activity of Y21pBpF/Y70pBpF/Y114pBpF was higher than that of the WT at a wide range of temperatures (35–80°C). Furthermore, the activity of Y21pBpF/Y70pBpF/Y114pBpF relative to that of the WT was temperature dependent, because the relative superiority of the activity increased with increasing temperatures (Figure 6C). The thermostability of Y21pBpF/Y70pBpF/Y114pBpF was higher than that of the WT at all temperatures tested in this study (Figure 6D). Furthermore, the SDS-PAGE analysis showed that the auto-degradation of Y21pBpF/Y70pBpF/Y114pBpF was slower than that of the WT when they were incubated at 65°C, suggesting that the mutations in mKerPA improved the autolytic resistance of the enzyme (Figure 6B). In addition, the Y21pBpF/Y70pBpF/Y114pBpF variant showed a significantly higher stability and activity under reducing conditions than that of the WT (Figures 6C,D).

The cleavage specificity and catalytic activity of Y21pBpF/Y70pBpF/Y114pBpF towards various protein substrates were determined and compared with those of the WT. As shown in Table 1, Y21pBpF/Y70pBpF/Y114pBpF showed improved activity in the hydrolysis of different complex substrates, and its intact feather degradation activity was approximately three times higher than that of the WT. Thus, the Y21pBpF/Y70pBpF/Y114pBpF variant developed in this study was more suitable for application in the keratin-rich waste recycling industry than the WT was. Using synthetic suc-AAPF-pNA as the substrate, the kinetic parameters of the WT and the Y21pBpF/Y70pBpF/Y114pBpF variant were determined (Table 2). The Y21pBpF/Y70pBpF/Y114pBpF variant displayed higher *K*_*m*_ and *k*_*cat*_ values than those of the WT, which indicated that the pBpF-based mutations in mKerPA decreased the substrate affinity and increased the turnover rate of the enzyme.

**TABLE 1:** Specific activities of wild-type (WT) enzyme and Y21pBpF/Y70pBpF/Y114pBpF variant on different protein substrates.

	Specific activity (U/mg)^a^	
Substrate	WT	Variant
Azocasein	(61.4 ± 4.2) × 10^3^	(85.6 ± 5.9) × 10^3^
Casein	(27.2 ± 2.1) × 10^3^	(40.8 ± 3.8) × 10^3^
BSA	(31.5 ± 1.7) × 10^3^	(39.1 ± 4.1) × 10^3^
Gelatine	(22.1 ± 3.6) × 10^3^	(26.2 ± 3.3) ×10^3^
Fibrin	(12.1 ± 3.1) × 10^3^	(15.7 ± 2.7) × 10^3^
Azocoll	(6.9 ± 0.7) × 10^3^	(8.1 ± 0.9) × 10^3^
Feather keratin	(4.3 ± 0.4) × 10^2^	(13.1 ± 0.7) × 10^2^
keratin azure	(1.3 ± 0.2) × 10^2^	(3.5 ± 0.3) × 10^2^
Intact feather	94 ± 3.7	261 ± 7.5

a Values are means ± standard deviation of three independent experiments. BSA, bovine serum albumin.

**TABLE 2:** Kinetic parameters and molecular masses of wild-type (WT) and Y21pBpF/Y70pBpF/Y114pBpF variant.

Enzyme	*K*_*m*_ (mM)^a^	*k*_*cat*_ (s^−1^)^a^	molecular mass
WT	0.57 ± 0.4	551 ± 26	32960.72
Variant	0.82 ± 0.7	731 ± 31	33224.90

a Values are means ± standard deviation of three independent experiments.

To confirm whether pBpF was incorporated into KerPA, a Quadrupole Time-of-Flight (QTOF)/MS analysis was conducted to determine the molecular masses of the WT and Y21pBpF/Y70pBpF/Y114pBpF variant. The successful incorporation of pBpF into the three sites (Y21, Y70, Y114) of KerPA in the Y21pBpF/Y70pBpF/Y114pBpF variant would be expected to yield a theoretical molecular mass 264.3 Da larger than that of the WT. The observed molecular masses of the WT and Y21pBpF/Y70pBpF/Y114pBpF variant determined using QTOF/MS analysis were 32960.72 and 33,224.90 Da, respectively (Table 2). Thus, the observed mass gain (264.18 Da) of the variant was consistent with the theoretical value, indicating that pBpF was properly incorporated into KerPA.

To determine whether the enhanced activity and thermostability induced by the pBpF substitution could be reproduced by the cAAs, the three sites Y21, Y70, and Y114 were independently mutated to nine other representative cAAs (Gly, Ala, Ser, Thr, Asp, Glu, Lys, Arg, and Gln). Among the 27 variants, only three, Y21R, Y21Q, and Y70E, matured properly and displayed remarkable azocaseinolytic activity (Supplementary Figures S3A,B). However, the stability of these three variants was significantly lower than that of the WT (Supplementary Figure S3C). Thus, pBpF substitution at Y21, Y70, Y114 sites in KerPA were irreplaceable in improving the enzyme properties.

### MD Analysis of the Variant Y21pBpF/Y70pBpF/Y114pBpF

**FIGURE 7:**
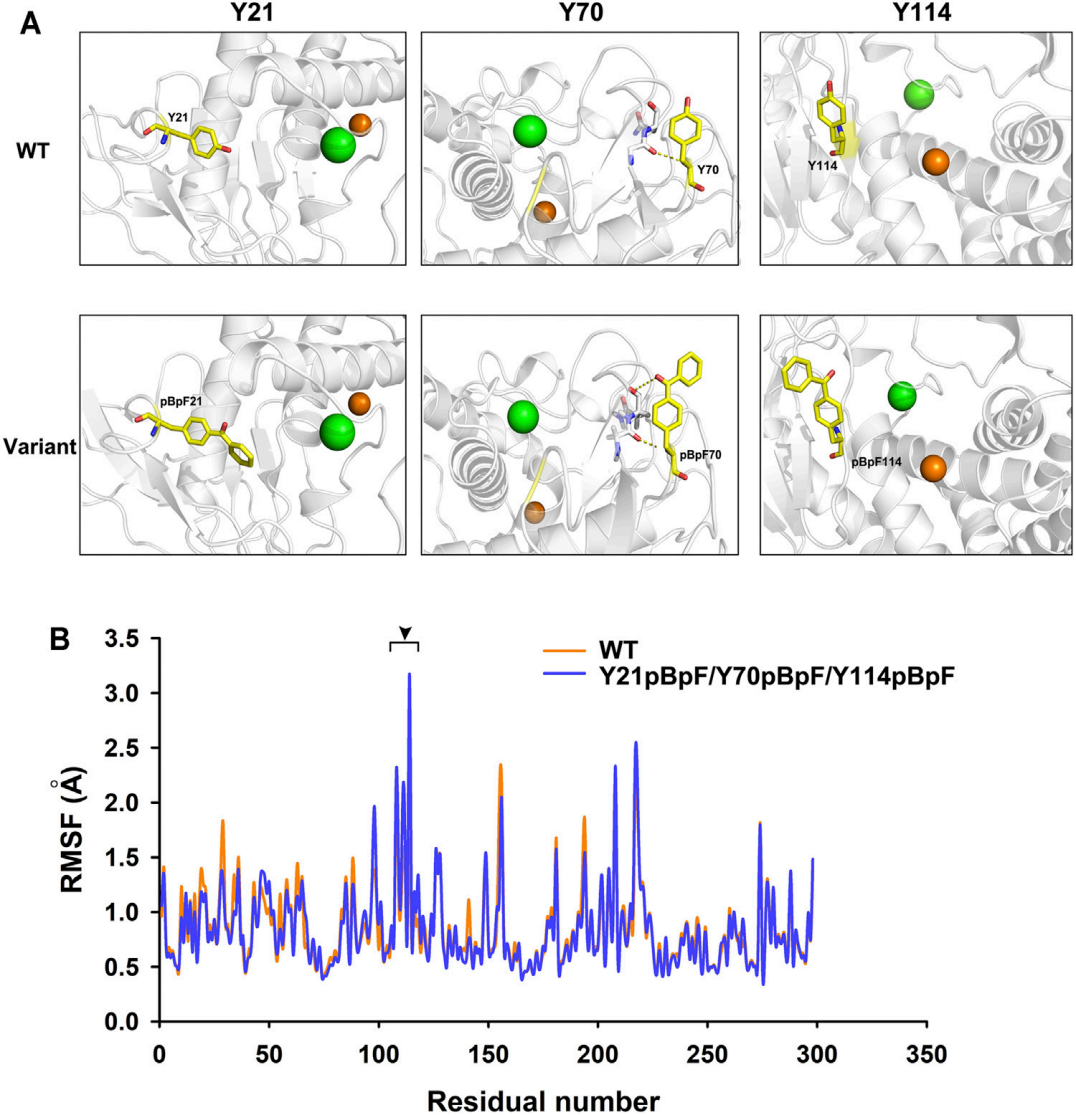
Structure and molecular dynamics analysis of local region of wild-type (WT) enzyme and its variants. **(A)** Local structures of WT and its variant. Green and orange balls in protein structure represent bound calcium and zinc ions, respectively. Mutant residues are shown as yellow sticks. Dotted lines represent interactions formed by original and substituted residues in WT enzyme and variants. **(B)** Residual root mean square fluctuation (RMSF) of keratinase from *Pseudomonas aeruginosa* (KerPA) and its variant. Black arrowhead indicates regions with increased residual flexibility in variant.

MD simulations of the Y21pBpF/Y70pBpF/Y114pBpF variant and WT were created using the Amber program. During simulation, the structures of the variant and the WT reached an equilibrium state, as reflected by the RMSD calculated for the Cα atoms. The overall RMSD values were similar between the variant and WT (Supplementary Figure S4). According to the stimulated structures of the WT, the Y21 residue of the WT was oriented towards the interior of the protein structure. Considered that better protein core packing is often linked to increased stability, substitution of Y21 with pBpF, which has bulkier side chains, may fill voids in the protein structure and thereby improve the thermostability of the enzyme (Figure 7A). The Y70 residue is located at the C-terminal of a long-loop structure and is oriented outside the WT structure. After replacing Y70 with pBpF, a new interaction was formed between the side chain of pBpF and the N9 residue, which may explain why the Y70pBpF mutation was beneficial to the thermostability of the enzyme (Figure 7A). The structure of the WT showed that the Y114 residue was located on a β-strand near the catalytic zinc ion. In the stimulated structure of the variant, a significant structural change can be observed in the Y114 located β-sheet contributing to the substrate-binding pocket (Supplementary Figure S5). The β-sheet was deformed and transformed into a flexible loop structure after sidechain modification of Y114, which may be favorable to the binding of substrates (Figure 7A). The loop structure is usually more flexible than the β-sheet, and the Y114pBpF mutation increased the flexibility of the catalytic site, as revealed by the local RMSF (Figure 7B). Increasing the flexibility of the active site of the enzyme is often linked to enhanced activity; thus, the MD analysis result might explain why substitution of Y114 with pBpF significantly improved the enzyme activity.

## Discussion

Keratinases that can degrade keratin-rich waste in an environmentally friendly manner have been the focus of increasing attention. To date, many keratinases from different microorganisms, including *Bacillus* sp., *Xanthomonas* sp., *Thermoactinomyces* spp., *Thermoanaerobacter* spp., *P. aeruginosa*, *Fervidobacterium islandicum*, *Meiothermus taiwanensis* WR-220, *Serratia marcescens*, and *Stenotrophomonas maltophilia*, have been isolated and characterised (Ho et al., 2013; Li, 2019). Most keratinases identified to date belong to the family of serine proteases with a few exceptions that are metalloproteases. Considerable progress has been made in the enhancement of the enzymatic properties of keratinases using protein engineering and directed evolution. After immobilisation onto a biotinylated solid matrix, the stability of KerA from *Bacillus licheniformis* PWD-1 was remarkably improved (Wang et al., 2003). The keratinolytic activity of thermophilic subtilisin WF146 from the thermophilic *Bacillus* sp. WF146 was enhanced by the modification of the autolytic sites and its thermostability was improved under reducing conditions (Liang et al., 2010). The catalytic efficiency and thermostability of keratinase KerSMD from *Stenotrophomonas* sp. were significantly improved by domain exchange with its homologous counterparts, KerSMF (Fang et al., 2016). Using computational aided rational design, the half-life of *B. licheniformis* BBE11-1 keratinase at 60°C increased 8.6-fold, and the ɑ-keratin digestion ability of the enzyme was also improved (Liu et al., 2013; Chen et al., 2014). The thermostability of keratinase from *Brevibacillus parabrevis* was remarkably enhanced by SDM at three positions (D181, Y217, S236) (Shi et al., 2018).

In nature, posttranslational modifications, including phosphorylation, methylation and glycosylation of specific residues are usually used to augment new chemistries in proteins, and regulate and enhance the function of proteins (Liu and Schultz, 2010). Thus, expanding the chemical diversity of the 20 cAAs could improve the protein properties. It has also been proven that introduction of ncAAs with different functional structures into proteins could enhance the physical, chemical, and biological properties of proteins (Xiao and Schultz, 2016). Although the enzymatic properties of keratinases have been improved mainly by cAA-based engineering, engineering keratinases with different ncAAs may greatly improve the ability to handle enzymatic structure and function. Among the methods developed for incorporation of ncAAs into proteins, genetic code expansion is superior because of its ability to incorporate various ncAAs site-specifically and at multiple sites into target proteins in all living organisms (Chin, 2014).

In this study, KerPA from *P. aeruginosa* was isolated and characterised, and evolved with 10 ncAAs by genetic code expansion. Several activities and thermostability enhanced variants were obtained, and the triple variant Y21pBpF/Y70pBpF/Y114pBpF was the best one when compared with the WT. It has been proposed that reducing agents are usually essential for keratinase to degrade keratin efficiently and the role of the reducing agents involved in breaking down the disulphide bonds and changing the conformation of keratin to expose proteolytic cleavage sites (Liang et al., 2010). Thus, enhancing the stability of keratinases under reducing conditions is of great significance for practical applications. Previously, the stability of keratinase KerBL from *Bacillus licheniformis* WHU under reducing conditions was improved by ncAA-based proximity-triggered chemical crosslinking (Yi et al., 2020). In this study, the activity and stability Y21pBpF/Y70pBpF/Y114pBpF were both improved compared with those of the WT under reducing conditions. This indicates that the Y21pBpF/Y70pBpF/Y114pBpF variant would be more suitable than the WT for application in the keratin-rich waste recycling industry.

The use of ncAAs in enzyme engineering and directed evolution may revolutionise the field. Specifically, ncAAs could provide protein engineers with powerful tools for introducing new chemical modifications into proteins and manipulating protein structures precisely at the single-atom level (Pagar et al., 2021). Many studies over the past decades have reported enhancement of the activity and stability of enzymes, as well as the creation of new functions by incorporating ncAAs (Pagar et al., 2021). For example, substitution of the peripheral active site residue Tyr309 with L-(7-hydroxycoumarin-4-yl)ethylglycine increased the activity of a bacterial phosphotriesterase (arPTE) by 8–11-fold (Ugwumba et al., 2011). Introducing a new hydrogen bond by mutating Tyr18 with m-chlorotyrosine remarkably improved the activity and stability of T4 lysozyme (Carlsson et al., 2018). The proof about the irreplaceability of ncAAs in enzyme engineering and evolution has also been occurring. For example, the introduction of para-aminophenylalanine at position 75 improved the activity of P450_BM3_ in oxidising (+)-nootkatone by 5-fold, which was not achieved with any cAAs (Cirino et al., 2003). Replacement of the proximal haem ligand His residue with Nδ-methyl histidine in myoglobin using genetic code expansion increased the haem redox potential and promiscuous peroxidase activity of the enzyme (Pott et al., 2018). Xiao *et al.* constructed a large ncAAs containing variant library of β-lactamase, and a variant V216AcrF (O-methyl-tyrosine) with improved activity was obtained after screening (Xiao et al., 2015). Their study demonstrated that this improvement could not be achieved by mutation of any other cAAs. In this study, it has also been found that the improved activity and thermostability of the Y21pBpF/Y70pBpF/Y114pBpF variant was not achieved by cAA mutations, which undoubtedly emphasises the critical role of ncAAs in enzyme engineering and evolution in some cases.

Notably, most studies on enzyme engineering and directed evolution with ncAAs by genetic code expansion describe only single-site substitutions with one specific ncAA (Pagar et al., 2021). In this study, the triple Y21pBpF/Y70pBpF/Y114pBpF variant displayed higher activity, stability or both than those of the single Y21pBpF, Y70pBpF, and Y114pBpF variants and the WT. This indicates that ncAAs could have accumulative effects on the properties of the enzyme when mutated at different sites. More than 200 ncAA-containing variants were screened in this study, and several variants with enhanced activity, stability, or both were identified. These variants had different ncAA mutations at various sites, and combining these mutations into one variant may further improve the properties of the enzyme. However, to achieve this goal, mutually orthogonal aminoacyl-tRNA synthetases/tRNA pairs for the incorporation of distinct ncAAs into one protein need to be identified and developed. The *Mj*TyrRS/tRNA and *P. horikoshii* lysyl-tRNA synthetase/tRNA pairs were previously proven to be mutually orthogonal and, therefore, could be used to simultaneously incorporate two different ncAAs into a single polypeptide (Chin, 2014). In addition, a set of triply orthogonal pyrrolysyl–tRNA synthetase/tRNA pairs for decoding three distinct ncAAs in a single polypeptide was recently developed by Dunkelmann *et al.* (Dunkelmann et al., 2020). Thus, it may conclude that simultaneously incorporating distinct ncAAs into proteins to improve enzyme properties could be achieved in the near future.

The main challenge to the broad application of ncAAs in enzyme engineering and directed evolution is that during protein expression, the medium requires supplementation with exogenous often expensive ncAAs, which would increase production cost. Autonomous biosynthesis of ncAAs using metabolic pathway engineering coupled with genetic code expansion *in situ* in host cells could avoid the need for exogenous supplementation with ncAAs and significantly reduce costs. Several efforts have been made to achieve that advantage in this field. p-Amino-phenylalanine, 5-hydroxytryptophan, L-phosphothreonine, L-dihydroxyphenylalanine, and S-allyl-L-cysteine were successfully biosynthesized in *E. coli* using enzymatic or metabolic pathway engineering, and were directly incorporated into the proteins using genetic code expansion (Exner et al., 2017; Zhang et al., 2017; Kim et al., 2018; Chen et al., 2020). The continuous progress in biochemistry, molecular biology, and synthetic biology will lead to the synthesis of an increasing number of ncAAs in engineered cells. Furthermore, further advances in ncAA mutagenesis techniques could render ncAAs more applicable in protein engineering and enzyme evolution.

## Data Availability

The original contributions presented in the study are included in the article/Supplementary Material, further inquiries can be directed to the corresponding author.
