# Supplementary material for: Enhancement of Activity and Thermostability of Keratinase From Pseudomonas aeruginosa CCTCC AB2013184 by Directed Evolution With Noncanonical Amino Acids
Source: Front Bioeng Biotechnol. 2021 Oct 18;9:770907. doi: 10.3389/fbioe.2021.770907 (PMC8558439; doi:10.3389/fbioe.2021.770907)
Supplement: Supplementary file 1 [file Table1.DOCX]

**TABLE S1. Oligonucleotide primers used in this study.**

| Primer | Oligonucleotide sequence * |
| --- | --- |
| **P1** | CTCAGTGGTGGTGGTGGTGGTG*CTCGAG*CAACGCGCTCGGGCAGGTCACGCCGACG |
| **P2** | CTTTAAGAAGGAGATATA*CATATG*GCCGACCTGATCGACGTGTCCAAACTCCCC |
| **P3** | GCATG*CCATGGA*TGCCGACCTGATCGACGTGTCCAAACTCCC |
| **P4** | CGCCC*AAGCTT*CAACGCGCTCGGGCAGGTCACGCC |
| **Y15TAG-F** | GGCAAGTAGAACTACGGCACCGACTACGGTCCGCTG |
| **Y15TAG-R** | GTAGTTCTACTTGCCGATCTTCTGGTTGCCGCCGGG |
| **Y17TAG-F** | GTACAACTAGGGCACCGACTACGGTCCGCTGATCGTC |
| **Y17TAG-R** | GGTGCCCTAGTTGTACTTGCCGATCTTCTGGTTGCCG |
| **Y21TAG-F** | CACCGACTAGGGTCCGCTGATCGTCAACGACCGCTG |
| **Y21TAG-R** | CGGACCCTAGTCGGTGCCGTAGTTGTACTTGCCGATC |
| **Y63TAG-F** | CAATACCTAGAAGCAGATCAATGGCGCCTACTCGCCGC |
| **Y63TAG-R** | CTGCTTCTAGGTATTGGTCGGGCAGGCGAAGCGGAACG |
| **Y70TAG-F** | GGCGCCTAGTCGCCGCTGAACGACGCGCATTTCTTC |
| **Y70TAG-R** | CGGCGACTAGGCGCCATTGATCTGCTTGTAGGTATTGG |
| **Y87TAG-F** | CAAGCTGTAGAAGGACTGGTTCGGCGCCAGCCCGC |
| **Y87TAG-R** | GTCCTTCTACAGCTTGAACACCACACCGCCGAAGAAATGC |
| **Y101TAG-F** | CAAGCTGTAGATGAAGGTGCACTACGGGCGCAGTG |
| **Y101TAG-R** | CTTCATCTACAGCTTGTGGGTCAGCGGGCTGGCGC |
| **Y106TAG-F** | GTGCACTAGGGGCGCAGTGTGGAGAACGCTTACTG |
| **Y106TAG-R** | GCGCCCCTAGTGCACCTTCATGTACAGCTTGTGGG |
| **Y114TAG-F** | GAACGCTTAGTGGGACGGTACGGCGATGCTGTTCG |
| **Y114TAG-R** | GTCCCACTAAGCGTTCTCCACACTGCGCCCGTAGTG |
| **Y130TAG-F** | CATGTTCTAGCCGCTGGTGTCGCTGGACGTGGCGG |
| **Y130TAG-R** | CAGCGGCTAGAACATGGTGGCGCCGTCGCCGAACAG |
| **Y155TAG-F** | CTGGTCTAGCGTGGCCAGTCCGGCGGGATGAACGAG |
| **Y155TAG-R** | GCCACGCTAGACCAGGCCGGAGTTCTGCTCGGTGAAG |
| **Y177TAG-F** | GAATTCTAGATGCGCGGCAAGAACGACTTCCTGATCGG |
| **Y177TAG-R** | GCGCATCTAGAATTCGGCCGCCTCGCCGGCCATGTC |
| **Y188TAG-F** | GATCGGCTAGGACATCAAGAAGGGCAGCGGTGCGC |
| **Y188TAG-R** | GATGTCCTAGCCGATCAGGAAGTCGTTCTTGCCGCGC |
| **Y199TAG-F** | CTGCGCTAGATGGACCAGCCCAGCCGTGACGGGCG |
| **Y199TAG-R** | GTCCATCTAGCGCAGCGCACCGCTGCCCTTCTTGATG |
| **Y216TAG-F** | GGGCAGTAGTACAACGGTATCGACGTGCACCATTCCAGC |
| **Y216TAG-R** | GTTGTACTACTGCCCGGCGTTGTCGATGGAACGCC |
| **Y217TAG-F** | CAGTACTAGAACGGTATCGACGTGCACCATTCCAGCGGC |
| **Y217TAG-R** | GATACCGTTCTAGTACTGCCCGGCGTTGTCGATGGAAC |
| **Y229TAG-F** | GGCGTGTAGAACCGCGCCTTCTACCTGCTGGCCAAC |
| **Y229TAG-R** | GCGGTTCTACACGCCGCTGGAATGGTGCACGTCGATAC |
| **Y234TAG-F** | GCCTTCTAGCTGCTGGCCAACTCGCCGGGCTGGG |
| **Y234TAG-R** | CAGCAGCTAGAAGGCGCGGTTGTACACGCCGCTGG |
| **Y257TAG-F** | CAACCGCTAGTACTGGACCGCGACCAGCACCTTCAAC |
| **Y257TAG-R** | CCAGTACTAGCGGTTGGCGTCGACGAACACCTCGAAG |
| **Y258TAG-F** | CGCTACTAGTGGACCGCGACCAGCACCTTCAACAG |
| **Y258TAG-R** | GGTCCACTAGTAGCGGTTGGCGTCGACGAACACCTC |
| **Y281TAG-F** | CGCAACTAGCCGGCGGCTGACGTCACTCGTGCCTTC |
| **Y281TAG-R** | CGCCGGCTAGTTGCGGTTCTGTGCCGAGCGGATCAC |
| **Y21G-F** | CACCGACGGCGGTCCGCTGATCGTCAACGACCGCTG |
| **Y21G-R** | CGGACCGCCGTCGGTGCCGTAGTTGTACTTGCCGATC |
| **Y21A-F** | CACCGACGCAGGTCCGCTGATCGTCAACGACCGCTG |
| **Y21A-R** | CGGACCTGCGTCGGTGCCGTAGTTGTACTTGCCGATC |
| **Y21S-F** | CACCGACAGCGGTCCGCTGATCGTCAACGACCGCTG |
| **Y21S-R** | CGGACCGCTGTCGGTGCCGTAGTTGTACTTGCCGATC |
| **Y21T-F** | CACCGACACCGGTCCGCTGATCGTCAACGACCGCTG |
| **Y21T-R** | CGGACCGGTGTCGGTGCCGTAGTTGTACTTGCCGATC |
| **Y21D-F** | CACCGACGACGGTCCGCTGATCGTCAACGACCGCTG |
| **Y21D-R** | CGGACCGTCGTCGGTGCCGTAGTTGTACTTGCCGATC |
| **Y21E-F** | CACCGACGAGGGTCCGCTGATCGTCAACGACCGCTG |
| **Y21E-R** | CGGACCCTCGTCGGTGCCGTAGTTGTACTTGCCGATC |
| **Y21K-F** | CACCGACAAGGGTCCGCTGATCGTCAACGACCGCTG |
| **Y21K-R** | CGGACCCTTGTCGGTGCCGTAGTTGTACTTGCCGATC |
| **Y21R-F** | CACCGACCGTGGTCCGCTGATCGTCAACGACCGCTG |
| **Y21R-R** | CGGACCACGGTCGGTGCCGTAGTTGTACTTGCCGATC |
| **Y21Q-F** | CACCGACCAGGGTCCGCTGATCGTCAACGACCGCTG |
| **Y21Q-R** | CGGACCCTGGTCGGTGCCGTAGTTGTACTTGCCGATC |
| **Y70G-F** | GGCGCCGGCTCGCCGCTGAACGACGCGCATTTCTTC |
| **Y70G-R** | CGGCGAGCCGGCGCCATTGATCTGCTTGTAGGTATTGG |
| **Y70A-F** | GGCGCCGCATCGCCGCTGAACGACGCGCATTTCTTC |
| **Y70A-R** | CGGCGATGCGGCGCCATTGATCTGCTTGTAGGTATTGG |
| **Y70S-F** | GGCGCCAGCTCGCCGCTGAACGACGCGCATTTCTTC |
| **Y70S-R** | CGGCGAGCTGGCGCCATTGATCTGCTTGTAGGTATTGG |
| **Y70T-F** | GGCGCCACCTCGCCGCTGAACGACGCGCATTTCTTC |
| **Y70T-R** | CGGCGAGGTGGCGCCATTGATCTGCTTGTAGGTATTGG |
| **Y70D-F** | GGCGCCGACTCGCCGCTGAACGACGCGCATTTCTTC |
| **Y70D-R** | CGGCGAGTCGGCGCCATTGATCTGCTTGTAGGTATTGG |
| **Y70E-F** | GGCGCCGAGTCGCCGCTGAACGACGCGCATTTCTTC |
| **Y70E-R** | CGGCGACTCGGCGCCATTGATCTGCTTGTAGGTATTGG |
| **Y70K-F** | GGCGCCAAGTCGCCGCTGAACGACGCGCATTTCTTC |
| **Y70K-R** | CGGCGACTTGGCGCCATTGATCTGCTTGTAGGTATTGG |
| **Y70R-F** | GGCGCCCGTTCGCCGCTGAACGACGCGCATTTCTTC |
| **Y70R-R** | CGGCGAACGGGCGCCATTGATCTGCTTGTAGGTATTGG |
| **Y70Q-F** | GGCGCCCAGTCGCCGCTGAACGACGCGCATTTCTTC |
| **Y70Q-R** | CGGCGACTGGGCGCCATTGATCTGCTTGTAGGTATTGG |
| **Y114G-F** | GAACGCTGGCTGGGACGGTACGGCGATGCTGTTCG |
| **Y114G-R** | GTCCCAGCCAGCGTTCTCCACACTGCGCCCGTAGTG |
| **Y114A-F** | GAACGCTGCATGGGACGGTACGGCGATGCTGTTCG |
| **Y114A-R** | GTCCCATGCAGCGTTCTCCACACTGCGCCCGTAGTG |
| **Y114S-F** | GAACGCTAGCTGGGACGGTACGGCGATGCTGTTCG |
| **Y114S-R** | GTCCCAGCTAGCGTTCTCCACACTGCGCCCGTAGTG |
| **Y114T-F** | GAACGCTACCTGGGACGGTACGGCGATGCTGTTCG |
| **Y114T-R** | GTCCCAGGTAGCGTTCTCCACACTGCGCCCGTAGTG |
| **Y114D-F** | GAACGCTGACTGGGACGGTACGGCGATGCTGTTCG |
| **Y114D-R** | GTCCCAGTCAGCGTTCTCCACACTGCGCCCGTAGTG |
| **Y114E-F** | GAACGCTGAGTGGGACGGTACGGCGATGCTGTTCG |
| **Y114E-R** | GTCCCACTCAGCGTTCTCCACACTGCGCCCGTAGTG |
| **Y114K-F** | GAACGCTAAGTGGGACGGTACGGCGATGCTGTTCG |
| **Y114K-R** | GTCCCACTTAGCGTTCTCCACACTGCGCCCGTAGTG |
| **Y114R-F** | GAACGCTCGTTGGGACGGTACGGCGATGCTGTTCG |
| **Y114R-R** | GTCCCAACGAGCGTTCTCCACACTGCGCCCGTAGTG |
| **Y114Q-F** | GAACGCTCAGTGGGACGGTACGGCGATGCTGTTCG |
| **Y114Q-R** | GTCCCACTGAGCGTTCTCCACACTGCGCCCGTAGTG |
| **D136A-F** | CGCCACGGCCAGCGACACCAGCGGATAGAACATGGTGG |
| **D136A -R** | GTCGCTGGCCGTGGCGGCCCACGAGGTCAGCCAC |
| **E172A-F** | GGCCGCCGCGCCGGCCATGTCGGAGAACGCCTCGTTCATC |
| **E172A -R** | GCCGGCGCGGCGGCCGAATTCTACATGCGCGGCAAGAACGAC |
| **E175A-F** | GTAGAATGCGGCCGCCTCGCCGGCCATGTCGGAGAACGCC |
| **E175A -R** | GCGGCCGCATTCTACATGCGCGGCAAGAACGACTTCCTGATCGGC |
| **D183A-F** | CAGGAAGGCGTTCTTGCCGCGCATGTAGAAT |
| **D183A -R** | AGAACGCCTTCCTGATCGGCTACGACATCAAGAAGG |
| **E172A/E175A-F** | GTAGAATGCGGCCGCCGCGCCGGCCATGTCGGAGAACGCCTCGTTCATC |
| **E172A/E175A-R** | GCCGGCGCGGCGGCCGCATTCTACATGCGCGGCAAGAACGACTTCCTG |
| **C30A-F** | GTCCATCTCGGCGCGGTCGTTGACGATCAGCGG |
| **C30A-R** | CGACCGCGCCGAGATGGACGATGGCAACGTC |
| **C58A -F** | GGTCGGGGCGGCGAAGCGGAACGGCGTGGAC |
| **C58A -R** | CGCTTCGCCGCCCCGACCAATACCTACAAGCAG |
| **C270A-F** | GATCACCCCGGCGGCGCCGCTGTTGAAGGTGCTGGTCGC |
| **C270A-R** | GCGGCGCCGCCGGGGTGATCCGCTCGGCACAGAACCG |
| **C297A -F** | CGCGCTCGGGGCGGTCACGCCGACGGTGCTGAAG |
| **C297A -R** | GGCGTGACCGCCCCGAGCGCGTTGCTCGAGCACCACC |

* Underlined sequences indicate the restriction enzyme sites used in this study. Red bases indicate the mutated codons.

**TABLE S2. Primer pairs, template and restriction enzyme sites used in the construction of expression plasmids.**

| Expression plasmid | Primer pair | Template | Restriction  enzyme site |
| --- | --- | --- | --- |
| pET22b-*KerPA* | P1+P2 | genome of *P. aeruginosa* | *Nde*I*-Xho*I |
| pET26b-*KerPA* | P3+P4 | genome of *P. aeruginosa* | *Nco*I*-Hind*III |
| pET26b-*KerPA_Y15TAG* | Y15TAG-F/R | pET26b-*KerPA* |  |
| pET26b-*KerPA_Y17TAG* | Y17TAG-F/R | pET26b-*KerPA* |  |
| pET26b-*KerPA_Y21TAG* | Y21TAG-F/R | pET26b-*KerPA* |  |
| pET26b-*KerPA_Y63TAG* | Y63TAG-F/R | pET26b-*KerPA* |  |
| pET26b-*KerPA_Y70TAG* | Y70TAG-F/R | pET26b-*KerPA* |  |
| pET26b-*KerPA_Y87TAG* | Y87TAG-F/R | pET26b-*KerPA* |  |
| pET26b-*KerPA_Y101TAG* | Y101TAG-F/R | pET26b-*KerPA* |  |
| pET26b-*KerPA_Y106TAG* | Y106TAG-F/R | pET26b-*KerPA* |  |
| pET26b-*KerPA_Y114TAG* | Y114TAG-F/R | pET26b-*KerPA* |  |
| pET26b-*KerPA_Y130TAG* | Y130TAG-F/R | pET26b-*KerPA* |  |
| pET26b-*KerPA_Y155TAG* | Y155TAG-F/R | pET26b-*KerPA* |  |
| pET26b-*KerPA_Y177TAG* | Y177TAG-F/R | pET26b-*KerPA* |  |
| pET26b-*KerPA_Y188TAG* | Y188TAG-F/R | pET26b-*KerPA* |  |
| pET26b-*KerPA_Y199TAG* | Y199TAG-F/R | pET26b-*KerPA* |  |
| pET26b-*KerPA_Y216TAG* | Y216TAG-F/R | pET26b-*KerPA* |  |
| pET26b-*KerPA_Y217TAG* | Y217TAG-F/R | pET26b-*KerPA* |  |
| pET26b-*KerPA_Y229TAG* | Y229TAG-F/R | pET26b-*KerPA* |  |
| pET26b-*KerPA_Y234TAG* | Y234TAG-F/R | pET26b-*KerPA* |  |
| pET26b-*KerPA_Y257TAG* | Y257TAG-F/R | pET26b-*KerPA* |  |
| pET26b-*KerPA_Y258TAG* | Y258TAG-F/R | pET26b-*KerPA* |  |
| pET26b-*KerPA_Y281TAG* | Y281TAG-F/R | pET26b-*KerPA* |  |
| pET22b-*KerPA_Y21G* | Y21G-F/R | pET22b-*KerPA* |  |
| pET22b-*KerPA_Y21A* | Y21A-F/R | pET22b-*KerPA* |  |
| pET22b-*KerPA_Y21S* | Y21S-F/R | pET22b-*KerPA* |  |
| pET22b-*KerPA_Y21T* | Y21T-F/R | pET22b-*KerPA* |  |
| pET22b-*KerPA_Y21D* | Y21D-F/R | pET22b-*KerPA* |  |
| pET22b-*KerPA_Y21E* | Y21E-F/R | pET22b-*KerPA* |  |
| pET22b-*KerPA_Y21K* | Y21K-F/R | pET22b-*KerPA* |  |
| pET22b-*KerPA_Y21R* | Y21R-F/R | pET22b-*KerPA* |  |
| pET22b-*KerPA_Y21Q* | Y21Q-F/R | pET22b-*KerPA* |  |
| pET22b-*KerPA_Y70G* | Y70G-F/R | pET22b-*KerPA* |  |
| pET22b-*KerPA_Y70A* | Y70A-F/R | pET22b-*KerPA* |  |
| pET22b-*KerPA_Y70S* | Y70S-F/R | pET22b-*KerPA* |  |
| pET22b-*KerPA_Y70T* | Y70T-F/R | pET22b-*KerPA* |  |
| pET22b-*KerPA_Y70D* | Y70D-F/R | pET22b-*KerPA* |  |
| pET22b-*KerPA_Y70E* | Y70E-F/R | pET22b-*KerPA* |  |
| pET22b-*KerPA_Y70K* | Y70K-F/R | pET22b-*KerPA* |  |
| pET22b-*KerPA_Y70R* | Y70R-F/R | pET22b-*KerPA* |  |
| pET22b-*KerPA_Y70Q* | Y70Q-F/R | pET22b-*KerPA* |  |
| pET22b-*KerPA_Y114G* | Y114G-F/R | pET22b-*KerPA* |  |
| pET22b-*KerPA_Y114A* | Y114A-F/R | pET22b-*KerPA* |  |
| pET22b-*KerPA_Y114S* | Y114S-F/R | pET22b-*KerPA* |  |
| pET22b-*KerPA_Y114T* | Y114T-F/R | pET22b-*KerPA* |  |
| pET22b-*KerPA_Y114D* | Y114D-F/R | pET22b-*KerPA* |  |
| pET22b-*KerPA_Y114E* | Y114E-F/R | pET22b-*KerPA* |  |
| pET22b-*KerPA_Y114K* | Y114K-F/R | pET22b-*KerPA* |  |
| pET22b-*KerPA_Y114R* | Y114R-F/R | pET22b-*KerPA* |  |
| pET22b-*KerPA_Y114Q* | Y114Q-F/R | pET22b-*KerPA* |  |
| pET22b-*KerPA_D136A* | D136A-F/R | pET22b-*KerPA* |  |
| pET22b-*KerPA_E172A* | E172A-F/R | pET22b-*KerPA* |  |
| pET22b-*KerPA_E175A* | E175A-F/R | pET22b-*KerPA* |  |
| pET22b-*KerPA_D183A* | D183A-F/R | pET22b-*KerPA* |  |
| pET22b-*KerPA_E172A/E175A* | E172A/E175A-F/R | pET22b-*KerPA* |  |
| pET22b-*KerPA_ E172A/E175A/D183A* | D183A-F/R | pET22b-*KerPA_E172A/E175A* |  |
| pET22b-*KerPA_D136A/ E172A/E175A/D183A* | D136A-F/R | pET22b-*KerPA_E172A/E175A/D183A* |  |
| pET22b-*KerPA_C30A* | C30A-F/R | pET22b-*KerPA* |  |
| pET22b-*KerPA_C58A* | C58A-F/R | pET22b-*KerPA* |  |
| pET22b-*KerPA_C270A* | C270A-F/R | pET22b-*KerPA* |  |
| pET22b-*KerPA_C297A* | C297A-F/R | pET22b-*KerPA* |  |
| pET22b-*KerPA_C30A/C58A* | C58A-F/R | pET22b-*KerPA_C30A* |  |
| pET22b-*KerPA_C270A/C297A* | C297A-F/R | pET22b-*KerPA_C270A* |  |

**Table S3. Amino acids compositions of mature KerPA**

| **Amino acid** | **Number** | **Mol(%)** | **Weight(%)** |
| --- | --- | --- | --- |
| **Ala** | 28 | 9.30 | 6.49 |
| **Cys** | 4 | 1.33 | 1.26 |
| **Asp** | 22 | 7.31 | 7.62 |
| **Glu** | 8 | 2.66 | 3.06 |
| **Phe** | 17 | 5.65 | 7.30 |
| **Gly** | 34 | 11.30 | 6.64 |
| **His** | 7 | 2.33 | 2.82 |
| **Ile** | 9 | 2.99 | 3.07 |
| **Lys** | 12 | 3.99 | 4.56 |
| **Leu** | 14 | 4.65 | 4.78 |
| **Met** | 9 | 2.99 | 3.49 |
| **Asn** | 20 | 6.64 | 6.87 |
| **Pro** | 11 | 3.65 | 3.29 |
| **Gln** | 8 | 2.66 | 3.04 |
| **Arg** | 14 | 4.65 | 6.34 |
| **Ser** | 23 | 7.64 | 6.29 |
| **Thr** | 17 | 5.65 | 5.27 |
| **Val** | 19 | 6.31 | 5.79 |
| **Trp** | 4 | 1.33 | 2.12 |
| **Tyr** | 21 | 6.98 | 9.90 |

**Figure S1. Activity screening of the crude enzyme samples of the ncAAs containing variants.**


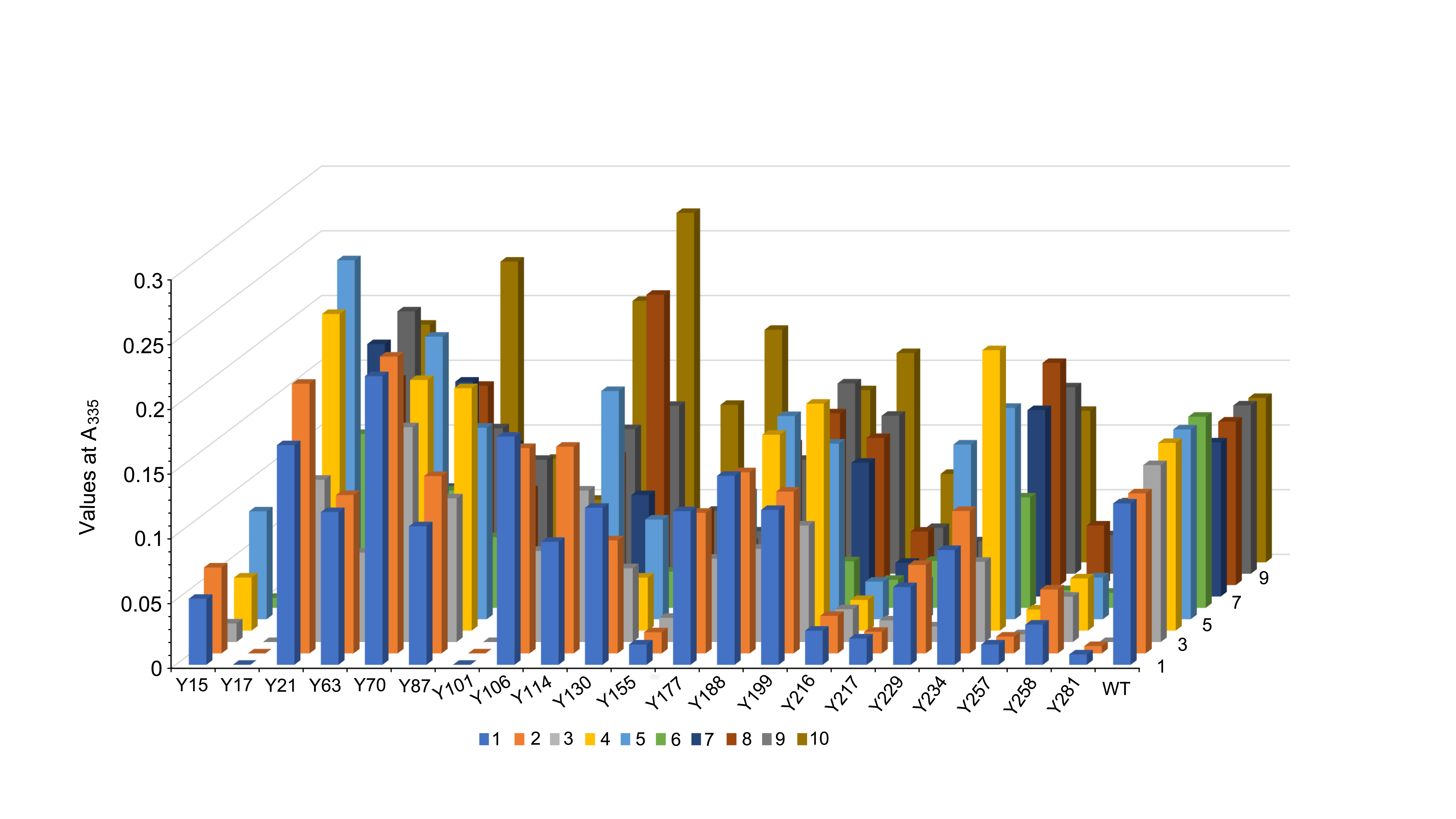


**Figure S2 Thermostability analysis of the crude enzyme samples of the ncAAs containing variants.**


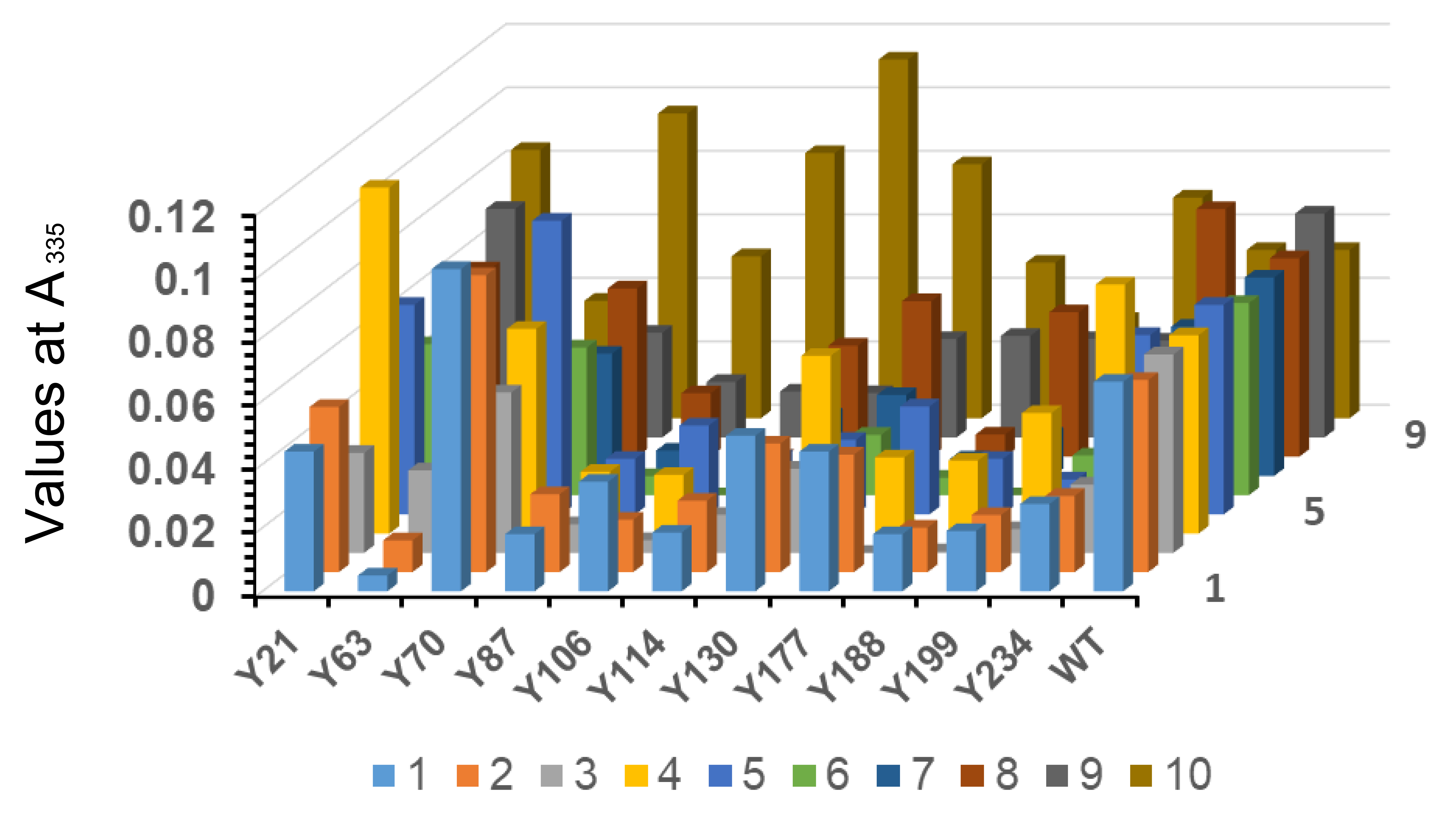

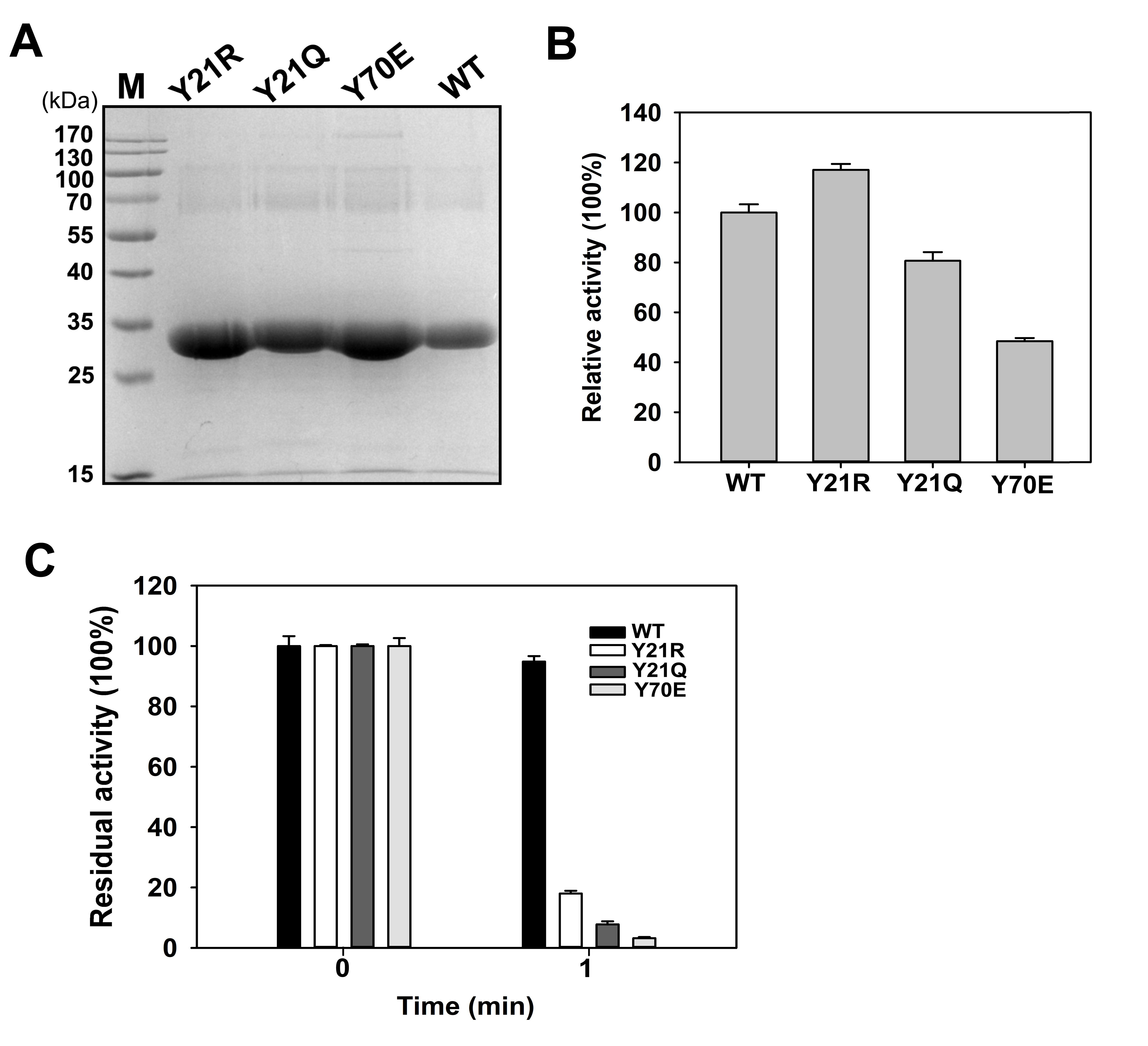


**Figure S3. Properties of the canonical amino acids containing variants.** (A) SDS-PAGE analysis of the purified samples of the vatiants. (B) Activity analysis of the variants. The azocaseinolytic activity assays were performed at 55℃ in PB buffer. (C) Thermostability analysis of the variants. The enzyme samples were incubated at 65℃ for 1min. and then performed standard activity assay using azocasein as substrate. Residual activity is expressed as a percentage of the original activity of each enzyme sample. The values are expressed as the means ± standard deviations (SDs) from three independent experiments.


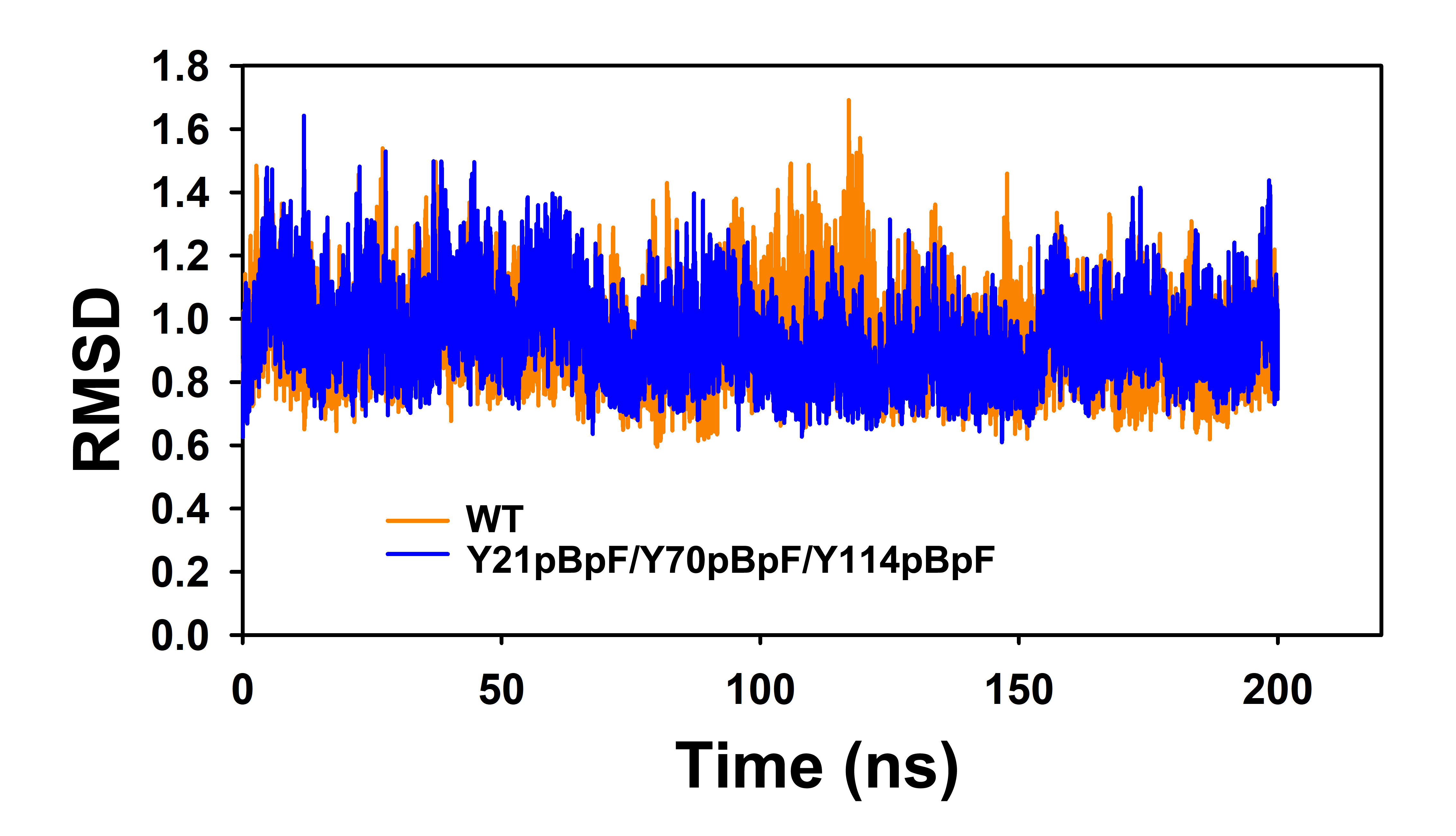


**Figure S4. RMSD values of the WT and its variant during 200 ns**


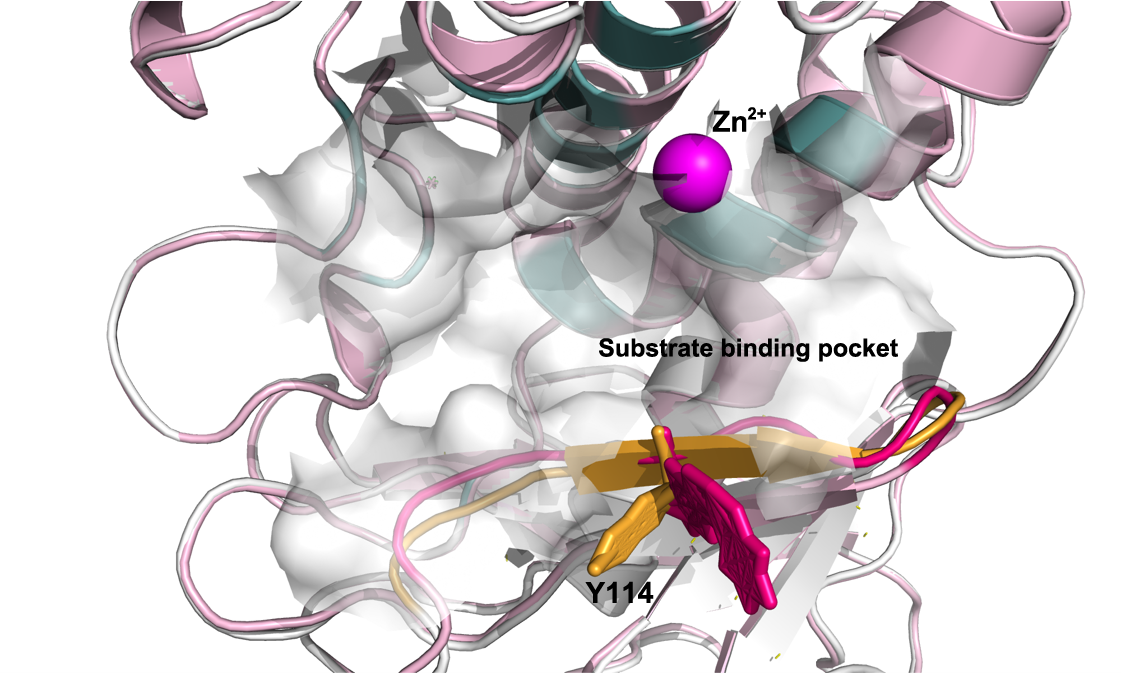


**Figure S5. Local structure alignment of the WT and its variant**
